# Enhancing the Detectable Chemical Space in an Effluent-Dominated Stream: Non-Target Analysis Reveals Potential Rapid *In Situ* Product Formation

**DOI:** 10.1021/acs.estlett.5c00509

**Published:** 2025-07-17

**Authors:** Alyssa L. Mianecki, Gregory H. LeFevre

**Affiliations:** § Department of Civil & Environmental Engineering, University of Iowa, 4105 Seamans Center, Iowa City, Iowa 52242, United States; ¶ IIHR-Hydroscience & Engineering, 100 C. Maxwell Stanley Hydraulics Laboratory, Iowa City, Iowa 52242, United States

**Keywords:** HRMS, nontarget analysis, effluent dominated streams, pharmaceuticals, pesticides, bioassays

## Abstract

Effluent-dominated streams are increasingly common in temperate regions and have potential for adverse ecological and human health effects. Nontarget analysis (NTA) using high resolution mass spectrometry (HRMS) is an emerging approach to examine complex chemical mixtures in the environment. Here, we leveraged archived samples previously analyzed for 154 target contaminants in a well-studied temperate region effluent-dominated stream and compared target results with suspected compounds from NTA. NTA enhanced the detectable chemical space by 20 times compared to target analysis alone. Target analysis was biased toward larger mass contaminants compared to the compounds detected with NTA, indicating that target analysis was not fully representative of the compound distribution for the effluent-impacted waters. Hierarchical cluster analysis exposed clusters of compounds significantly upregulated at the effluent/stream mixing zone, revealing evidence of a novel phenomenon wherein transformation product and/or metabolite formation appears to occur rapidly *in situ*. Additionally, an exposure-driven NTA retrospective analysis uncovered upregulation of 7endocrine-disrupting compounds that may explain prior bioassay results. These findings have urgent implications for ecosystems and downstream communities experiencing de facto wastewater reuse conditions. NTA offers enhanced characterization of complex mixtures in effluent-dominated streams and can reveal novel mixture dynamics otherwise masked when employing target analysis alone.

## Graphical abstract

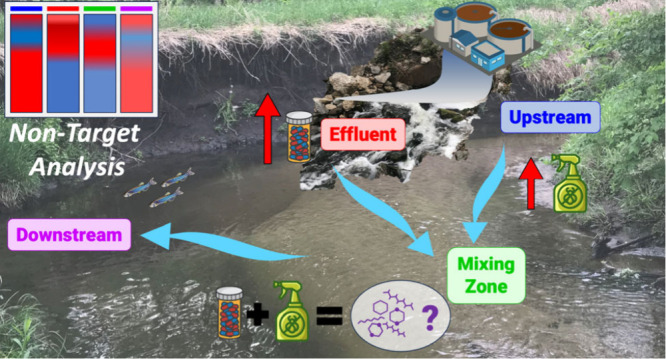

## Introduction

Polar organic contaminants (e.g., pesticides, pharmaceuticals, corrosion inhibitors, per- and polyfluoroalkyl substances [PFAS]) in aquatic environments exist as complex mixtures with potential to cause adverse impacts to aquatic biota and downstream communities through chronic exposure.^1,2^ Wastewater effluent-dominated streams (EDSs), which are increasingly common worldwide, represent worst-case contaminant exposure conditions for aquatic ecosystems and downstream communities.^3^ A nationwide study reported that 50% of drinking water treatment plants, which serve approximately 82% of the U.S. population, are potentially impacted by de facto wastewater reuse.^4^ Additionally, small EDSs contain elevated concentration exposure conditions for aquatic biota due to limited dilution and concurrent nonpoint source exposure routes.^5^ The mixtures of contaminants in EDSs have the potential for synergistic and additive interactions in aquatic biota and cause adverse effects like endocrine disruption, behavior changes, and altered genotypic/phenotypic expression.^6–8^ Our understanding of complex mixtures and their effects is limited because small bench-scale studies often fail to elicit the same responses as field-based studies.^9–14^ This discrepancy arises in part from the inherent limitations of targeted methods used to measure contaminants in the source water, as these targeted methods only allow for the analysis of preselected compounds.

Complex mixtures of polar organic contaminants cannot be fully characterized using targeted chemical studies alone. Previously, we developed a long-term study site at a temperate-region EDS (1 site upstream, 3 sites downstream of wastewater treatment plant) to characterize the spatiotemporal occurrence/fate^15,16^ and risk dynamics^5^ of target pharmaceuticals, neonicotinoids,^17^ and known metabolites. The wastewater treatment plant (WWTP) contributes significant chemical/flow inputs to the stream, with total contaminant concentrations decreasing with distance downstream and mixture composition evolution primarily driven by differential attenuation of individual chemicals via sorption to the bed sediments.^15,16^ Yet, concurrent biological analyses demonstrated effects that were unexplained by effluent-derived contaminants measured with target analysis. For example, fathead minnows exposed *in situ* up- and downstream of the WWTP outfall exhibited significant differential gene expression.^18^ Additionally, the BLYES bioassay revealed that the highest level of estrogenicity occurred upstream of the WWTP, where there is no link to effluent-derived contaminants.^19^ These findings indicate that the comprehensive complex mixtures in the effluent-dominated stream may have greater biological impacts than individual contaminants, and that targeted methods alone were insufficient to reveal the contaminant sources.

Comprehensive nontarget analysis (NTA) and suspect screening using high-resolution mass spectrometry (HRMS) are emerging tools for characterizing complex contaminant mixtures that have potential to uncover new perspectives on fundamental mixture dynamics and their connections to biological end points. Effect-driven analysis integrates biological assays to prescreen suspect samples before HRMS analysis and is considered the ‘gold standard’ for connecting mixtures to biological pathways and effects.^20^ Nevertheless, effect-driven analysis is resource-intensive, requires full predesign, and cannot leverage archived samples for which contextual data may exist. In contrast, exposure-driven approaches can enrich existing data sets when samples of known biological significance are screened with follow-up HRMS.^21^ Retrospective NTA studies are extremely valuable for adding resolution to samples of known importance, thereby enhancing the detectable chemical space for existing data sets.^22^ Although the chemical space boundaries are determined during the initial sample collection, processing, and analysis^22^ (e.g., choice of extraction methods, liquid vs gas chromatography), retrospective analysis with NTA can enhance the resolution of the existing chemical space to probe novel connections with target chemical/bioassay data or reveal otherwise masked trends. Therefore, the overall objective of this research was to apply a retrospective, exposure-driven NTA approach to enhance the detectable chemical space to (1) characterize the attenuation and product formation dynamics within the complex mixture beyond the capabilities of the prior targeted data sets, and (2) evaluate unknown contaminants that may drive known biological responses (i.e., estrogenicity bioassays, RNA-Seq results). Using NTA, we discovered a novel phenomenon wherein transformation products form rapidly in the effluent/receiving water mixing zone—which challenges our prior assumption that sorption was the primary driver of differential attenuation.^16^ Additionally, this research has urgent implications for de facto wastewater reuse because the intermediates formed could have important ecological or human health effects for downstream communities.

## Materials and Methods

### Chemicals

A list of chemicals and solvents is provided in .

### Study Reporting Tool

The NTA Study Reporting Tool (SRT) was used in preparation for this manuscript^23^ and is available as Supporting Information.

### Site Description, Sample Processing, and Analysis

Muddy Creek is a well-studied effluent-dominated stream in the temperate ecoregion of east-central Iowa (Figure S1).^5,15–19,24–26^ Archived sample extracts from previous studies were selected for retrospective analysis based on prior target data and exposure relevance (Section S1 **)**. Samples were collected sequentially with streamflow (though not strictly Lagrangian) at four established USGS monitoring sites in January 2018, May 2018, July 2019, and August 2020 during baseflow conditions: (1) *US1*–100 m upstream from WWTP, (2) *EFF*–WWTP outfall, (3) *DS1*–100m downstream from WWTP outfall, and (4) *DS2*–5.1km downstream of WWTP outfall (Figure S1, Table S1). Full details on sample processing, analysis, and QA/QC are in Section S2.

### Data Analysis

Compound Discoverer (CD) version 3.3.3.200 (Thermo Scientific) was used to process raw files (full details: Section S2). Initial peak picking tolerances were set at a minimum mass of 5 ppm, minimum peak intensity of 500,000 counts, and minimum S/N ratio of 3. Background peaks were removed (max sample/blank ratio = 5), peak areas were normalized (constant median), and compound names were assigned using predicted compositions, mass list searches (internal list with 154 compounds and imported NORMAN^27^ list), mzCloud searches, and ChemSpider searches. Remaining CD parameters are listed in Table S5. Data were further postprocessed to filter suspect compounds to approximately Schymanski Level 2–3 Confidence^28^ (Figure S3; tentative candidates with predicted matches, name matches, and MS2 matches) before performing hierarchical cluster analysis (HCA). MS2 spectra matches for named example compounds in the text/figures are shown in Section S3. Further statistical analysis to compare suspect compounds with target compounds^15,17^ was performed in GraphPad Prism 9.0.0.

## Results and Discussion

### Nontarget Analysis Enhances the Detectable Chemical Space of Complex Mixtures

Nontarget analysis (NTA) enhances the detectable chemical space^22^ compared to target analysis, especially in complex environmental mixtures. To evaluate chemical space differences in this effluent-dominated stream (EDS), we compared detection frequencies of quantified contaminants to suspected compounds. Quantified contaminants included detections from a targeted list of 154 pharmaceuticals, pesticides, and PFAS that were previously measured in the EDS and reported by the USGS (Section S2).^29,30^ We measured 30/154 contaminants with targeted analysis at US1, 93/154 at EFF, 88/154 at DS1, and 83/154 at DS2. In comparison, we revealed 641 suspect compounds at US1, 2045 at EFF, 1713 at DS1, and 1562 at DS2 using NTA. Overall, we enhanced the detectable chemical space by roughly 20-times using suspect NTA methods at each site compared to comprehensive target analysis. Chemical fingerprints generated by HRMS NTA are a standard and increasingly common approach for characterizing complex mixtures,^31^ revealing comparative trends in molecular weight and polarity (Figure 1). Interestingly, both targeted and NTA data sets yielded a 3-fold increase in WWTP-derived compounds compared the background water (US1), followed by a 1-fold decrease in compounds downstream.

**Figure 1:**
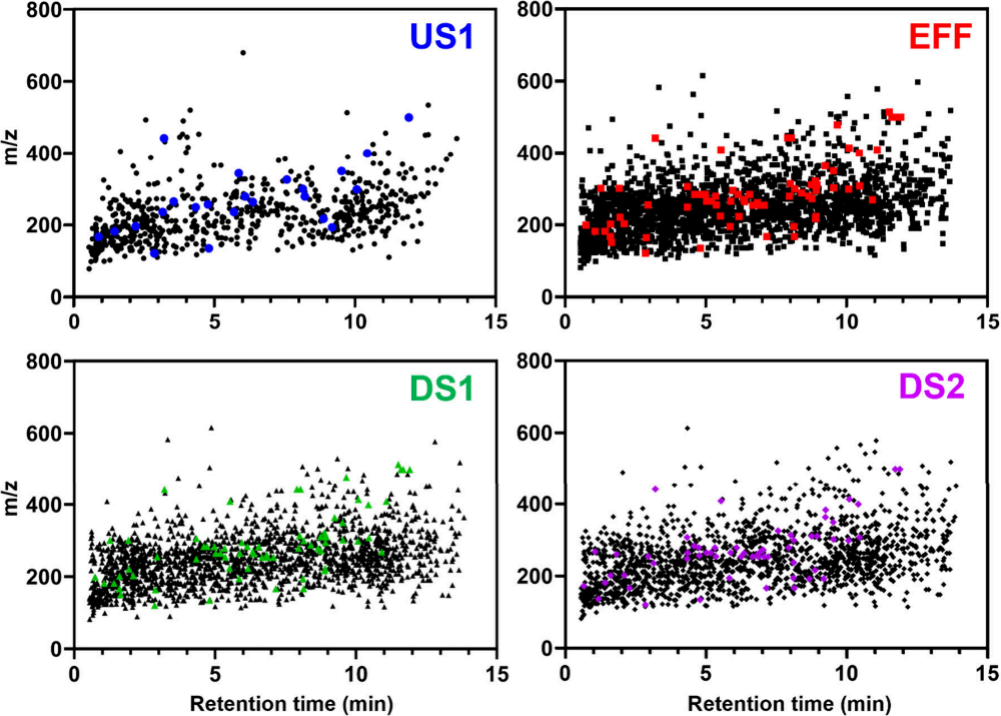
Fingerprint diagrams (*m*/*z* vs retention time) of suspected compounds at each site. The black shapes represent the broad suite of named suspect compounds detected with our NTA method (see Figure S3 for detailed description). For named suspect compounds (black shapes) there were *n* = 641 at US1, *n* = 2045 at EFF, *n* = 1713 at DS1, and *n* = 1562 at DS2. The colored shapes represent the smaller suite of suspect compounds that can be matched to the 154 USGS target chemicals (see Figure S4 for detailed description). For possible USGS mass list matches (colored shapes), there were *n* = 23 at US1, *n* = 70 at EFF, *n* = 56 at DS1, and *n* = 59 at DS2. Note: the colored shapes are not targeted LC-MS/MS results—the targeted results cannot be concurrently represented on this figure due to different retention times [RT] between methods. These fingerprint diagrams illustrate the difference in chemical space covered by broad suspect analysis compared to more selective analysis suite (mirroring target analysis compounds) at the four sites of the effluent-dominated stream study site, Muddy Creek, Johnson County, Iowa (US1 = 0.1km upstream of effluent outfall, EFF = effluent, DS1 = 0.1km downstream of effluent outfall, DS2 = 5.1km downstream of effluent outfall).

The mass distributions of nontarget compounds, compared to those of target contaminants, provide valuable insights into the analyzed chemical space. We compared the distributions between target contaminants (monoisotopic masses) and suspect compounds (*m*/*z*) for all sites in Muddy Creek (Figure 2), following established methods to avoid redundancy from multiple adducts or ions. Prior literature reports these approaches yield comparable results within acceptable error tolerances for large data sets.^32,33^ At US1, the mass distributions between target and suspect compounds were not significantly different (Kolmogorov–Smirnov test: *p* = 0.5728). In contrast, at sites EFF, DS1, and DS2 that are heavily impacted by wastewater, the mass distributions were significantly different between target and suspect compounds at each site (Kolmogorov–Smirnov test: EFF *p* < 0.0001; DS1 *p* = 0.0003; DS2 *p* = 0.0035). At sites impacted by effluent, mean compound masses were smaller in suspect distributions than the target distributions (also Figure S6). These results indicate that the target compound distributions do not fully represent the chemical space of the effluent-dominated stream, as even a large target suite (*n* = 154) was not a representative subsample of the chemical space population of the NTA data, and may be biased toward higher-mass contaminants. Lower molecular weight chemicals are often more mobile^34^ in the environment; therefore, NTA can better characterize and enhance this chemical space for mixtures in effluent-dominated streams. When the broader NTA chemical space was included (Figure S7), the relative concentrations of suspect compounds in Muddy Creek remained relatively constant downstream of the WWTP, implying more conservative transport of the total contaminant mixture compared to the attenuation of select target compounds observed in prior work.^15,16^

**Figure 2:**
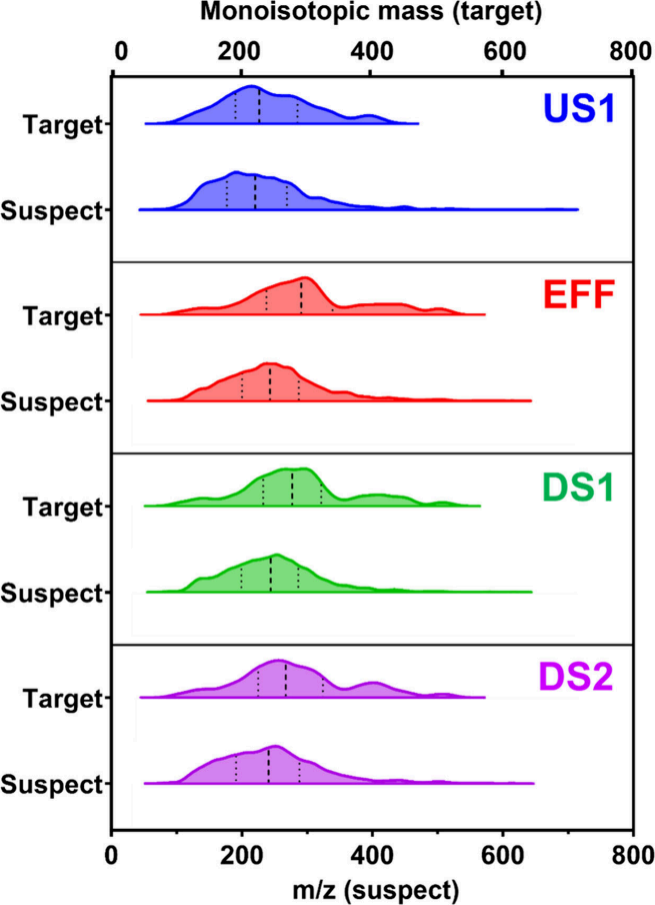
Mass distributions of previously published targeted contaminants (LC-MS/MS, monoisotopic mass) compared to mass distributions of suspect compounds (LC-Orbitrap HRMS, *m*/*z*) for each site at Muddy Creek (Johnson Country, Iowa, USA; US1 = 0.1km upstream of effluent outfall, EFF = effluent, DS1 = 0.1km downstream of effluent outfall, DS2 = 5.1km downstream of effluent outfall). In this study, we used monoisotopic mass (±1 *m*/*z*) and *m*/*z* (±5 ppm) as equivalent comparisons between the target and suspect data sets because these values have been established to fall within an acceptable error tolerance of each other.^32,33^ Dashes indicate medians and dots indicate lower and upper quartiles. Targeted compounds include pharmaceuticals, pesticides, and PFAS that were previously detected and reported by the USGS^29,30^ between 2018 and 2020 (*n*_US1_ = 30; *n*_EFF_ = 93; *n*_DS1_ = 88; *n*_DS2_ = 83). Suspected compounds from our retrospective LC-HRMS method are reported here (*n*_US1_ = 641; *n*_EFF_ = 2045; *n*_DS1_ = 1713; *n*_DS2_ = 1562). At US1, the target and suspect contaminant distributions are not significantly different (Kolmogorov–Smirnov Test: *p* = 0.5728). Nevertheless, the target and suspect contaminant distributions are statistically different for EFF, DS1, and DS2 (Kolmogorov–Smirnov Tests: EFF *p* < 0.0001; DS1 *p* = 0.0003; DS2 *p* = 0.0035).

### Nontarget Analysis Reveals Potential *In Situ* Product Formation

Clusters of significantly upregulated compounds occurred in the mixing zone downstream of the source water and wastewater effluent, suggesting that potential transformation products formed quickly in the EDS. We evaluated spatial patterns of suspect compounds using hierarchical clustering analysis (HCA), expecting to observe two distinct clusters of contaminants: one cluster indicative of source water and another cluster relating to effluent-impacted water that attenuated from the source, with dilution of the effluent downstream. Instead, HCA (*n* = 2114 suspect compounds) revealed distinct upregulated clusters of compounds at all four sites in Muddy Creek (Figure 3). Upstream of the effluent zone (US1), 813 compounds were upregulated (Cluster 1), including pesticides and corrosion inhibitors likely from nonpoint sources (e.g., atrazine, 4-/5-methyl-benzotriazole; MS2 spectra matches: Figures S11–S14). At the effluent outfall (EFF) there were 966 upregulated compounds (Cluster 2), including many common municipal wastewater contaminants like pharmaceuticals (*e*.*g.*, lidocaine, venlafaxine, trimethoprim; MS2: Figures S15–S20). The source water and treated effluent mixed thoroughly within 100 m of downstream travel, reaching DS1 in 8 min.^16^ At DS1, a cluster of 180 upregulated compounds (Cluster 3) imply transformation products and potentially novel metabolites formed rapidly in the mixing zone. Additionally, an upregulated cluster of 55 features (Cluster 4) at DS2 was identified (e.g., PFBS, DEET, azithromycin, fluconazole; MS2: Figures S27–S30), located 5km (11 h) downstream of the WWTP.^16^ The presence of upregulated clusters downstream of the WWTP mixing zone was unexpected and the incidence of known transformation products has human and ecosystem exposure implications for other wastewater-impacted systems.

**Figure 3:**
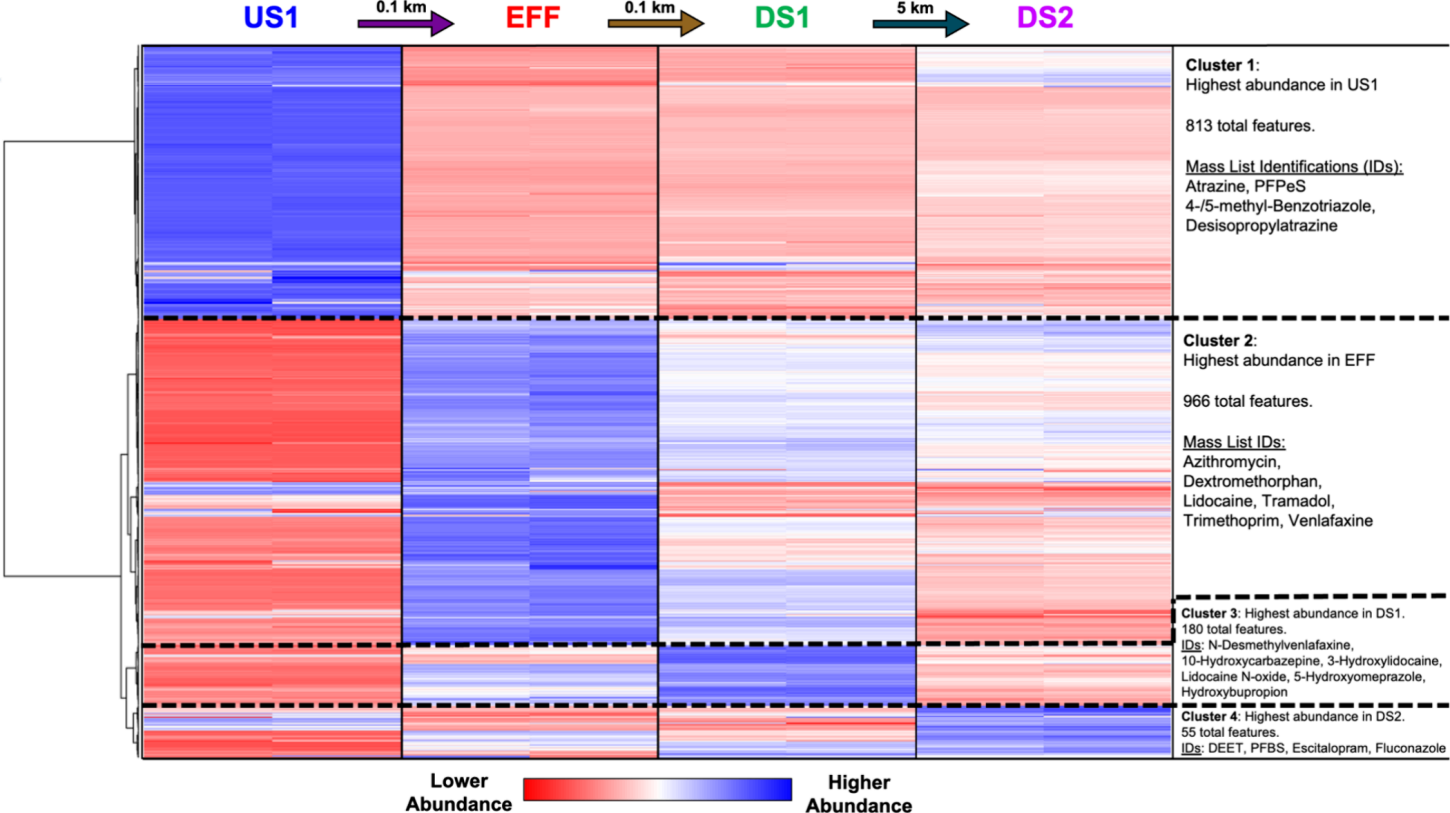
Hierarchical cluster analysis (HCA) of archived surface water samples in Muddy Creek from July 2019, constrained to 2114 filtered suspect compounds with names. HCAs for January 2018, May 2018, and August 2020 are presented in Figures S8–S10. Clusters of significantly upregulated suspect compounds are identified on the right, with included example Mass List IDs representing example upregulated suspect features from the given cluster identified via MS2 spectral library matching (Figures S11–S30). Columns represent the four sampling sites in Muddy Creek (US1 = 0.1km upstream of effluent outfall, EFF = effluent, DS1 = 0.1km downstream of outfall, DS2 = 5.1km downstream of outfall), with duplicate injections shown. Horizontal lines represent individual chemical features arranged by similarity, denoted by the dendrogram. Color represents peak area abundance (absent = dark red, increasing to light red, white, light blue, and dark blue with increasing peak area). The HCA was created with Compound Discoverer (v3.3) with peak areas normalized to medians. The clustering parameters include Ward’s linkage method and Euclidean distancing. Dark blue clusters (i.e., Cluster 1, Cluster 2, etc.) highlight groupings of compounds upregulated based on relative peak area response for sites along the stream reach.

Suspected transformation products appeared to form quickly in the mixing zone. Chemicals in DS1/Cluster 3 were distinct from the EFF/Cluster 2, even with the short distance and travel time between sites. In Cluster 3, multiple known pharmaceutical metabolites were identified, including N-desmethylvenlafaxine, 10-hydroxycarbazepine, 3-hydroxylidocaine, lidocaine N-oxide, 5-hydroxyomeprazole, and hydroxybupropion (MS2 spectra matching: Figures S21–S26). From targeted analysis,^15,16^ we know pharmaceutical parent compounds along with their metabolites (e.g., venlafaxine/desvenlafaxine, metformin/guanylurea) are present in the municipal effluent discharge following treatment processes. It is unsurprising to observe some metabolites present in the wastewater effluent following biological treatment in the activated sludge plant.^15,16^ These parent/metabolite pairs differentially attenuate during movement down the stream reach after being discharged to surface water, with attenuation thought to be driven by sorption/interactions with the bed sediments.^15,16,35^ Presence of transformation products in Cluster 3 implies rapid *in situ* compound transformation that was previously overlooked; however, we lack specific evidence for a driving mechanism particularly at the time scales relevant. Phototransformation can occur rapidly; with half-lives of <1 h for some pharmaceuticals in sunlit streams.^36–38^ DOM or nitrate/nitrite in the source water could act as indirect photosensitizers.^37^ The narrow/circumneutral pH range across sites (7.5–7.9 from US1→EFF→DS1) makes acid/base-catalyzed hydrolysis reactions unlikely. Biodegradation is also improbable due to slower rates and low microbial abundance in the water column;^39^ however, microbial communities in wastewater-impacted streams are complex.^40^ Interactions with DOM or inorganic components (e.g., flocculation under varying ionic strengths) could produce distinct features in the mixing zone.

Cluster 3 represents compounds, including parent compounds and transformation products, that are significantly more abundant at DS1. These compounds may also be present at other sites; however, their relative enrichment at DS1 highlights distinct chemical processes occurring in the mixing zone. Although sampling followed streamflow, sampling was not strictly Lagrangian; higher-resolution sampling in future studies could improve process understanding. The goal of HCA was to reveal spatial patterns in NTA feature relatedness at a defined filtering level (see Materials and Methods and SI), not to achieve complete compound identification. Specifically named compounds, identified via MS2 spectral library matching (Section S3), are presented as representative examples within each cluster. Further investigation of clusters could support future retrospective analyses. We highlight results from July 2019 as an illustrative case due to relevant summer temperature conditions and chemicals profiles observed in previous studies.^15^ Similar patterns indicative of *in situ* transformation were observed on other dates, though less pronounced (Figures S8–S10). Muted patterns on other dates may reflect more variable hydrologic or biogeochemical conditions. Overall, NTA revealed a cluster of upregulated compounds in the effluent mixing zone, suggesting rapid product formation not captured by previous target analyses. This, along with differential attenuation and variable inputs, likely contributes to complex mixture exposures with important implications for organisms in effluent-dominated streams.

### Retrospective Exposure-Driven Approach Revealed Stronger Nonpoint Influence Upstream of WWTP

Nontarget analysis revealed multiple previously overlooked potential endocrine-disrupting compounds in the source water. We originally hypothesized that WWTP-derived pharmaceuticals would drive estrogenicity in the effluent-dominated stream.^15^ Unexpectedly, the highest estrogenicity (E2Eq) was measured at US1 (Figure S31),^19^ a site unaffected by effluent.^15^ Because E2eq did not correlate with target contaminants,^19^ we retrospectively analyzed archived samples using NTA as an exposure-driven approach. Our method revealed seven named significantly upregulated (*p* < 0.05, Log_2_ Fold Change >2 based on raw peak area) suspected endocrine disrupting compounds (EDCs) associated with high E2eq at US1 (Figure S32, Table S9). Suspected compounds included pesticides like atrazine (herbicide, MS2 spectra library matching: Figure S33) and siduron (herbicide, MS2: Figure S34). Atrazine, a known endocrine disruptor linked to intersex effects in fish,^41–44^ was previously measured in Muddy Creek through targeted analysis.^15,25^ Siduron, a suspected endocrine disruptor^45,46^ related to diuron was also measured.^25^ Benzisothiazolinone (1,2-benzisothiazolin-3-one) is a biocide common in stormwater runoff with known thyroid-disrupting effects in fish (MS2: Figure S35).^47–49^ Crystal violet (gentian violet) is a synthetic dye in biological applications and fertilizers, and was another identified suspect EDC (MS2: Figure S36).^50–52^ Suspected hormones included 19-norandrosterone and 5α-dihydrotestosterone, both endogenous androgens (MS2: Figures S37, S38);^53,54^ and melengestrol, a synthetic cattle growth promoter (MS2: Figure S39).^55,56^ Pesticides and hormones from nonpoint runoff may therefore contribute more to estrogenicity in effluent-dominated streams than previously indicated by targeted analysis.^57^

Research concerning biological effects of contaminants in EDSs often focuses on a few known wastewater-derived compounds, such as antidepressants, while overlooking the broader contaminant mixture.^58,59^ For example, previous research by our team found significant transcriptomic changes across multiple biological pathways in fathead minnows () exposed to Muddy Creek surface water that were not observed when fish were exposed to chemicals individually^18,19^ —supporting the need to further characterize contaminant mixtures. NTA alone cannot definitively link suspect compounds to biological effects; however, retrospective NTA of samples with known biological relevance combined with targeted analysis can help identify potential connections. We recommend that future studies integrate effect-directed analysis with emerging big data approaches, such as machine learning, to better link transcriptomic responses to NTA results and other large omics data sets, enhancing predictive power and biological relevance.

### Environmental Implications

Previous research at Muddy Creek asserted differential attenuation of compound mixtures was primarily driven by interactions with the streambed sediments.^15,16^ We identified a potentially novel, rapid transformation phenomenon not captured by target analysis, signaling more complex spatiotemporal dynamics in mixture compositions. These findings have critical and urgent implications for de facto wastewater reuse because downstream communities relying on effluent-impacted source waters may be exposed to both persistent parent compounds and numerous unknown metabolites or disinfection byproduct precursors.^60^ Target analyses do not accurately represent the total contaminant exposure mixture profile, as they are limited to lists of known contaminants,^15,16,35^ which can mask critical attenuation and product formations. Retrospective NTA studies, such as this work, enhance the resolution of the detectable chemical space and add value to important archived samples. Nevertheless, retrospective analysis cannot expand the total chemical space because sample collection, extraction, and chromatography have already been completed, which is an inherent limitation.^22^ Retrospective studies are also constrained by the original sampling design; future research could benefit from higher-resolution spatiotemporal or Lagrangian sampling. Additionally, complex mixture matrix effects may vary spatially/temporally, influencing generated HRMS signals. Despite these limitations, NTA and targeted analysis are complementary, each offering useful data dimensionality that can be applied iteratively to understand environmental processes. Indeed, NTA is not a replacement for target analysis. It is important to recognize that data processing and filtering choices, whether by software or analysts, can impact NTA interpretation. For example, stricter thresholds for peak quality rating or the signal-to-noise ratios may improve data quality but risk excluding relevant compounds. Finally, although NTA cannot directly link suspect endocrine-disrupting compounds to biological effects, NTA serves as an important tool for guiding further investigation by identifying potential chemical sources and connections to biological outcomes through exposure-driven analysis. By bridging advances in analytical chemistry and molecular biology, retrospective NTA supports the long-term efforts to enhance the prediction of toxicological responses to complex and evolving environmental mixtures.

## Supplementary Material





## Notes

Site and sample descriptions, supporting analytical methods and chemicals, data processing, supplemental figures and statistics, and supporting references (PDF)
NTA Study Reporting Tool (SRT) used in the preparation of this manuscript (XLSX)
