# Supplementary material for: Enhancing the Detectable Chemical Space in an Effluent-Dominated Stream: Non-Target Analysis Reveals Potential Rapid In Situ Product Formation
Source: Environ Sci Technol Lett. 2025 Jul 17;12(8):1038–45. doi: 10.1021/acs.estlett.5c00509 (PMC12351525; doi:10.1021/acs.estlett.5c00509)
Supplement: Supplementary file 1 [file ez5c00509_si_001.pdf]

Supporting Information

Enhancing the Detectable Chemical Space in an Effluent-Dominated Stream: Non-Target Analysis Reveals Potential Rapid *in situ* Product Formation

Alyssa L. Mianecki[§¶] and Gregory H. LeFevre[§¶*]

[§]Department of Civil & Environmental Engineering, University of Iowa, 4105 Seamans Center, Iowa City, IA 52242, United States; [¶]IIHR-Hydroscience &Engineering, 100 C. Maxwell Stanley Hydraulics Laboratory, Iowa City, IA 52242, United States

<u>CONTAINS</u>: site and sample descriptions, supporting analytical methods and chemicals, data processing, supplemental figures and statistics, supporting references.

60 total pages (inclusive of this page) with 39 supporting figures and 9 supporting tables.

Section S1: Site and Sample Descriptions

Muddy Creek

Muddy Creek is located between the municipalities of North Liberty and Coralville, IA, USA (Latitude 41°42'00", Longitude 91°33'46"). It is a small, low-order stream with a drainage area of 22.5 km^2 and is impacted by both agricultural and urban land use. The North Liberty wastewater treatment plant (WWTP) discharges treated effluent from the city of North Liberty into the creek. The WWTP is designed with a membrane bioreactor to remove particles >0.004 µm. There are 4 established USGS monitoring sites along the stream reach: **US1** (0.1 km upstream of the WWTP outfall, USGS 05454050), **EFF** (effluent outfall, USGS 05454051), **DS1** (0.1 km downstream of the effluent outfall, USGS 05454052), and **DS2** (5 km downstream of the effluent outfall, USGS 05454090 flow gage) and samples have been collected at all 4 sites since 2017 (**Figure S1**).

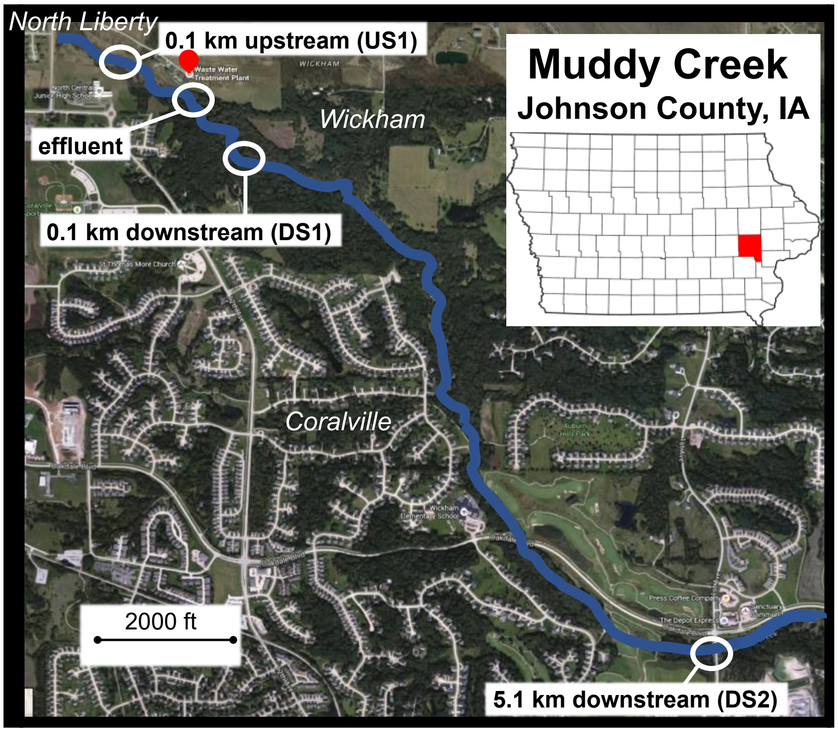

Figure S1. Map of Muddy Creek sampling sites within the cities of North Liberty and Coralville, Iowa, USA[1,2]. The red balloon indicates the location of the North Liberty WWTP.

Archived Samples

Four sets of samples from Muddy Creek were previously collected in January 2018, May 2018, July 2019, and August 2020 at the four established USGS monitoring sites (16 samples total) (**Figure S1**). Also see **Sample Analysis. Water** samples for this retrospective analysis were previously fortified with a deuterated standard mixture (n=8 surrogates, **Section S2**), extracted using Waters HLB solid phase extraction (SPE) cartridges then eluted with 1:1 acetonitrile:water and spiked with $^{\text{13C-caffeine}}$ as an internal standard, and reported for target pharmaceuticals1 and neonicotinoids2 (full details in below). This previously-established method demonstrated surrogate recoveries >85% (86-103%) for compounds over a wide range of chemical properties.[1] Preserved extracts in 1:1 acetonitrile:water were sealed in 2 mL amber glass vials and stored at -20°C. For this work, extracts were analyzed (2×polarity switching full scan MS1, 75-750 *m/z*; reinjected 1×positive and 1×negative data-dependent MS/MS [ddMS2], 75-750 *m/z* to confirm identifications (**Table S2-S4**) with a Thermo Vanquish Flex UHPLC (Agilent Poroshell 120, EC-C18 2.7 µm, 2.1×100) coupled to a Thermo Q Exactive hybrid quadrupole Orbitrap mass spectrometer. Solvent blanks were analyzed every 15 samples (no carry-over observed in blanks), and method blanks (1 per date, deionized water) were extracted/analyzed alongside samples. Multiple of the originally-spiked surrogates (*i.e.*, bupropion-d9, imidacloprid-d4, venlafaxine-d6, citalopram-d6) were detected in all NTA sample extracts, a qualitative indication check of confirmed presence that archived samples demonstrated longevity of at least the added compounds. A QC mixture of 18 pharmaceuticals/pesticides was analyzed to aid in relative retention time (RRT) rank-order matching to an internal mass list (**Table S6**), wherein the RRT was applied as a rank-order manual screen (*i.e.*, ensure consistent relative elution times) to eliminate duplicate identification of compounds that eluted at different times.

Data Analysis. We used Compound Discoverer™ (CD) version 3.3.3.200 (Thermo Scientific), a small molecule structure identification software to process raw files. The data processing workflow (**Figure S2, Table S5**) was originally developed from a pre-existing CD workflow (Environmental w Stats Unknown ID w Online and Local Database Searches) and further refined using literature input.[6] Initial peak picking tolerances were set at a minimum mass of 5 ppm, minimum peak intensity of 500,000 counts, and minimum S/N ratio of 3. Background peaks were removed (max sample/blank ratio=5), peak areas were normalized (constant median), and compound names were assigned using predicted compositions, mass list searches (internal list with 154 compounds and imported NORMAN7 list), mzCloud searches, and ChemSpider searches. The remaining CD parameters are listed in **Table S5.** Data were further post-processed to filter suspect compounds to approximately Schymanski Level 2-3 Confidence8 (**Figure S3**; tentative candidates with predicted matches, name matches, and MS2 matches) before hierarchical cluster analysis (HCA; Euclidean distances calculated from log-normalized peak areas; Ward's clustering method). Fold-change and up-/down-regulation analysis was based on unadjusted peak area analysis. For any specifically 'named' compounds discussed as highlighted examples in the text or figures, MS2 library spectra matching results (*i.e.*, mzCloud library match) are presented in the SI. Further statistical analysis to compare suspect compounds with target compounds1,2 was performed in GraphPad Prism 9.0.0.

Chemicals and Target Data for more information. These four sets of samples were analyzed by the USGS for 154 compounds including pharmaceuticals, pesticides, and PFAS (**Table S1**), and the samples were analyzed at UIowa for 21 pharmaceuticals and neonicotinoids (**Table S1**). At UIowa, the samples were originally extracted with Waters HLB SPE cartridges (with the exception

of one group from August 2020, which used Phenomenex Strata X-CW SPE cartridges) and analyzed for 21 target compounds consisting of common pharmaceuticals and neonicotinoid insecticides, and were subsequently reported.[1,2] The water samples are data rich and contain at least 2 overlapping datasets (**Table S1**).

Table S1. Muddy Creek surface water samples re-analyzed with HRMS have various overlapping datasets as outlined below.

Sample Set	USGS Pharmaceuticals/ pesticides (121 total compounds)[3]	USGS PFAS (33 total compounds)	UIowa pharmaceuticals/ neonicotinoids (21 total compounds)[1,2]	Estrogen equivalents (E2Eq) [4]	Gene isoforms from fish[5]	Bioconcentrations (fish/insects/spiders)
January 2018	✗	✗	✗	✗		
May 2018	✗	✗	✗	✗		
July 2019	✗		✗		✗	
August 2020	✗	✗	✗			✗

Section S2: Analytical Methods and Chemicals

Sample Analysis. Water samples for this retrospective analysis were previously fortified with a deuterated standard mixture (n=8 surrogates, **Section S2**), extracted using Waters HLB solid phase extraction (SPE) cartridges then eluted with 1:1 acetonitrile:water and spiked with ^{13}C-caffeine as an internal standard, and reported for target pharmaceuticals[1] and neonicotinoids[2] (full details in below). This previously-established method demonstrated surrogate recoveries >85% (86-103%) for compounds over a wide range of chemical properties.[1] Preserved extracts in 1:1 acetonitrile:water were sealed in 2 mL amber glass vials and stored at -20°C. For this work, extracts were analyzed (2×polarity switching full scan MS1, 75-750 *m/z*; reinjected 1×positive and 1×negative data-dependent MS/MS [ddMS2], 75-750 *m/z* to confirm identifications (**Table S2-S4**) with a Thermo Vanquish Flex UHPLC (Agilent Poroshell 120, EC-C18 2.7 µm, 2.1×100) coupled to a Thermo Q Exactive hybrid quadrupole Orbitrap mass spectrometer. Solvent blanks were analyzed every 15 samples (no carry-over observed in blanks), and method blanks (1 per date, deionized water) were extracted/analyzed alongside samples. Multiple of the originally-spiked surrogates (*i.e.*, bupropion-d_9, imidacloprid-d_4, venlafaxine-d_6, citalopram-d_6) were detected in all NTA sample extracts, a qualitative indication check of confirmed presence that archived samples demonstrated longevity of at least the added compounds. A QC mixture of 18 pharmaceuticals/pesticides was analyzed to aid in relative retention time (RRT) rank-order matching to an internal mass list (**Table S6**), wherein the RRT was applied as a rank-order manual screen (*i.e.*, ensure consistent relative elution times) to eliminate duplicate identification of compounds that eluted at different times.

Data Analysis. We used Compound Discoverer™ (CD) version 3.3.3.200 (Thermo Scientific), a small molecule structure identification software to process raw files. The data processing workflow

(**Figure S2, Table S5**) was originally developed from a pre-existing CD workflow (Environmental w Stats Unknown ID w Online and Local Database Searches) and further refined using literature input.[6] Initial peak picking tolerances were set at a minimum mass of 5 ppm, minimum peak intensity of 500,000 counts, and minimum S/N ratio of 3. Background peaks were removed (max sample/blank ratio=5), peak areas were normalized (constant median), and compound names were assigned using predicted compositions, mass list searches (internal list with 154 compounds and imported NORMAN[7] list), mzCloud searches, and ChemSpider searches. The remaining CD parameters are listed in **Table S5**. Data were further post-processed to filter suspect compounds to approximately Schymanski Level 2-3 Confidence[8] (**Figure S3**; tentative candidates with predicted matches, name matches, and MS2 matches) before hierarchical cluster analysis (HCA; Euclidean distances calculated from log-normalized peak areas; Ward's clustering method). Fold-change and up-/down-regulation analysis was based on unadjusted peak area analysis. For any specifically 'named' compounds discussed as highlighted examples in the text or figures, MS2 library spectra matching results (*i.e.*, mzCloud library match) are presented in the SI. Further statistical analysis to compare suspect compounds with target compounds[1,2] was performed in GraphPad Prism 9.0.0.

Chemicals and Target Data Repositories

Full information on target contaminants and solid phase extraction methods were published by Hui Zhi et al[1] and Webb et al.[2]

<u>Open source published USGS data is also available at the following:</u>

- Water-quality data for a pharmaceutical study at Muddy Creek in North Liberty and Coralville, Iowa, 2017-2018 [9]

- Water-quality data for a statewide assessment of per- and polyfluoroalkyl substances (PFAS) study in Iowa, 2019-2020 [10]
- Data release for August 2020 is currently pending with USGS, target pharmaceutical and pesticide data were reported to us directly from the lab.

Every sample was previously spiked with a mixture of isotopically labeled compounds that represent a range of chemical properties (purchased from Sigma-Aldrich, St. Louis, MO):

1. imidacloprid-d_4 (CAS 1015855-75-0)
2. metformin-d_6 (CAS 1185166-01-1)
3. venlafaxine-d_6 (CAS 1062606-12-5)
4. bupropion-d_9 (CAS 1189725-26-5)
5. carbamazepine-d_{10} (CAS 132183-78-9)
6. citalopram-d_6 (CAS 1190003-26-9)
7. 1H-benzotriazole-d_4 (CAS 1185072-03-0)

Note: Because metformin was not effectively retained on HLB SPE cartridges (only Strata X-CW), we did not see or expect to see any detection of metformin-d_6 during non-target analysis.

And the samples were spiked with an internal standard solution mixture:

1. caffeine-$^{13}C_3$ (CAS 78072-66-9)
2. thiamethoxam-d_3 (CAS 1294048-82-0)

All solutions were prepared in methanol and stored at -20°C. Other solvents used were acetonitrile, water, and formic acid were all optima LC-MS grade (Fisher, Fair Lawn, NJ).

Chromatography Parameters

Table S2. Chromatography parameters used for non-target analysis. A Thermo Vanquish Flex UHPLC system was fitted with an Agilent C18 column system for peak separations before mass spectrometry. Guard column: Agilent Eclipse Plus C18, 2.1×5 mm, 1.8 µm. Analytical column: Agilent Poroshell 120, EC-C18 2.7 µm, 2.1×100 (PN 695775-902(T)).

<table>
<tr><th colspan="7">UPLC Settings</th></tr>
<tr><td colspan="2">Pump</td><td rowspan="11"></td><td colspan="4"><u>Flow Gradient</u></td></tr>
<tr><td colspan="2"><u>General Settings</u></td><td>Time</td><td>Flow [mL/min]</td><td>% B</td><td>Curve</td></tr>
<tr><td colspan="2">Solvents</td><td>0.000</td><td colspan="3">Run</td></tr>
<tr><td>% A1</td><td>Water with 0.1% Formic</td><td>0.000</td><td>0.400</td><td>10.0</td><td>5</td></tr>
<tr><td>% A2</td><td>Water</td><td>4.000</td><td>0.400</td><td>20.0</td><td>5</td></tr>
<tr><td>% B1</td><td>ACN with 0.1% Formic</td><td>8.000</td><td>0.400</td><td>40.0</td><td>5</td></tr>
<tr><td>% B2</td><td>Acetonitrile</td><td>10.000</td><td>0.400</td><td>60.0</td><td>5</td></tr>
<tr><td colspan="2"></td><td>15.000</td><td>0.400</td><td>90.0</td><td>5</td></tr>
<tr><td colspan="2">Pressure Limits (psi)</td><td>15.200</td><td>0.400</td><td>10.0</td><td>5</td></tr>
<tr><td>Lower Limit</td><td>0</td><td>20.000</td><td>0.400</td><td>10.0</td><td>5</td></tr>
<tr><td>Upper Limit</td><td>9000</td><td>20.000</td><td colspan="3">Stop Run</td></tr>
</table>

Mass Spectrometry Parameters

Table S3. Mass spectrometry was performed in succession with the UPLC listed above. The HRMS setup was a Thermo Q Exactive hybrid quadrupole Orbitrap mass spectrometer. Each sample was injected in duplicate with this polarity switching full scan method.

**Polarity Switching - Full Scan**					
Method of Q Exactive		***Setup***		***Experiments***	
**Overall method settings**		**Tunefiles**		**Full MS – SIM**	
**Global Settings**		**General**		**General**	
Use lock masses	off	Switch Count	0	Runtime	0 to 15 min
Lock mass injection	–	Base Tunefile C:\Xcalibur\methods\300ul-min_020122.mstune		Polarity	positive
Chrom. peak width (FWHM)	3 s	**Contact Closure**		In-source CID	0.0 eV
**Time**		**General**		**Full MS – SIM**	
Method duration	20.00 min	Used	FALSE	Microscans	1
**Customized Tolerances (+/-)**		Start in Closed	TRUE	Resolution	70,000
Lock Masses	–	Switch Count	0	AGC target	1e6
Inclusion	10.00 ppm	**Syringe**		Maximum IT	200 ms
Exclusion	–	**General**		Number of scan ranges	1
Neutral Loss	–	Used	FALSE	Scan range	75 to 750 m/z
Mass Tags	–	Start in OFF	TRUE	Spectrum data type	Profile
Dynamic Exclusion	–	Stop at end of run	FALSE	**Full MS – SIM**	
		Switch Count	0	**General**	
		**Pump setup**		Runtime	0 to 15 min
		Syringe type	Hamilton	Polarity	negative
		Flow rate	3.000 µL/min	In-source CID	0.0 eV
		Inner diameter	2.303 mm	**Full MS – SIM**	
		Volume	250 µL	Microscans	1
		**Divert Valve A**		Resolution	70,000
		**General**		AGC target	1e6
		Used	TRUE	Maximum IT	200 ms
		Start in 1-2	TRUE	Number of scan ranges	1
		Switch Count	1	Scan range	75 to 750 m/z
		**Element 1**		Spectrum data type	Profile
		At	15.00 min		
		Switches to	1-6		

Table S4. Mass spectrometry was performed in succession with the UPLC listed above. The HRMS setup was a Thermo Q Exactive hybrid quadrupole Orbitrap mass spectrometer. Each sample was injected once in positive mode and once in negative mode ddMS2.

Positive or Negative ddMS2

Method of Q Exactive	
**Overall method settings**	
**Global Settings**	
Use lock masses	off
Lock mass injection	–
Chrom. peak width (FWHM)	3 s
**Time**	
Method duration	20.00 min
**Customized Tolerances (+/-)**	
Lock Masses	–
Inclusion	10.00 ppm
Exclusion	–
Neutral Loss	–
Mass Tags	–
Dynamic Exclusion	–

Experiment	
**Full MS / dd-MS² (TopN)**	
**General**	
Runtime	0 to 15 min
Polarity	positive or negative
In-source CID	0.0 eV
Default charge state	1
Inclusion	–
Exclusion	–
Tags	–
**Full MS**	
Microscans	1
Resolution	70,000
AGC target	1.00E+06
Maximum IT	200 ms
Number of scan ranges	1
Scan range	75 to 750 m/z
Spectrum data type	Profile
**dd-MS² / dd-SIM**	
Microscans	1
Resolution	17,500
AGC target	1.00E+05
Maximum IT	50 ms
Loop count	3

Setup	
**Tunefiles**	
**General**	
Switch Count	0
Base Tunefile C:\Xcalibur\methods\300ul-min_020122.mstune	
**Contact Closure**	
**General**	
Used	FALSE
Start in Closed	TRUE
Switch Count	0
**Syringe**	
**General**	
Used	FALSE
Start in OFF	TRUE
Stop at end of run	FALSE
Switch Count	0
**Pump setup**	
Syringe type	Hamilton
Flow rate	3.000 µL/min
Inner diameter	2.303 mm
Volume	250 µL
**Divert Valve A**	
**General**	
Used	TRUE

MSX count	1
TopN	3
Isolation window	1.0 m/z
Isolation offset	0.0 m/z
Scan range	200 to 2000 m/z
Fixed first mass	–
(N)CE / stepped (N)CE	nce: 20, 40, 60
Spectrum data type	Centroid
**dd Settings**	
Minimum AGC target	4.00E+03
Intensity threshold	8.00E+04
Apex trigger	–
Charge exclusion	–
Multiple charge states	all
Peptide match	–
Exclude isotopes	on
Dynamic exclusion	3.0s
If idle ..	pick others

Start in 1-2	TRUE
Switch Count	1
**Element 1**	
At	15.00 min
Switches to	1-6

Section S3: Data Processing

Compound Discoverer Workflow Parameters

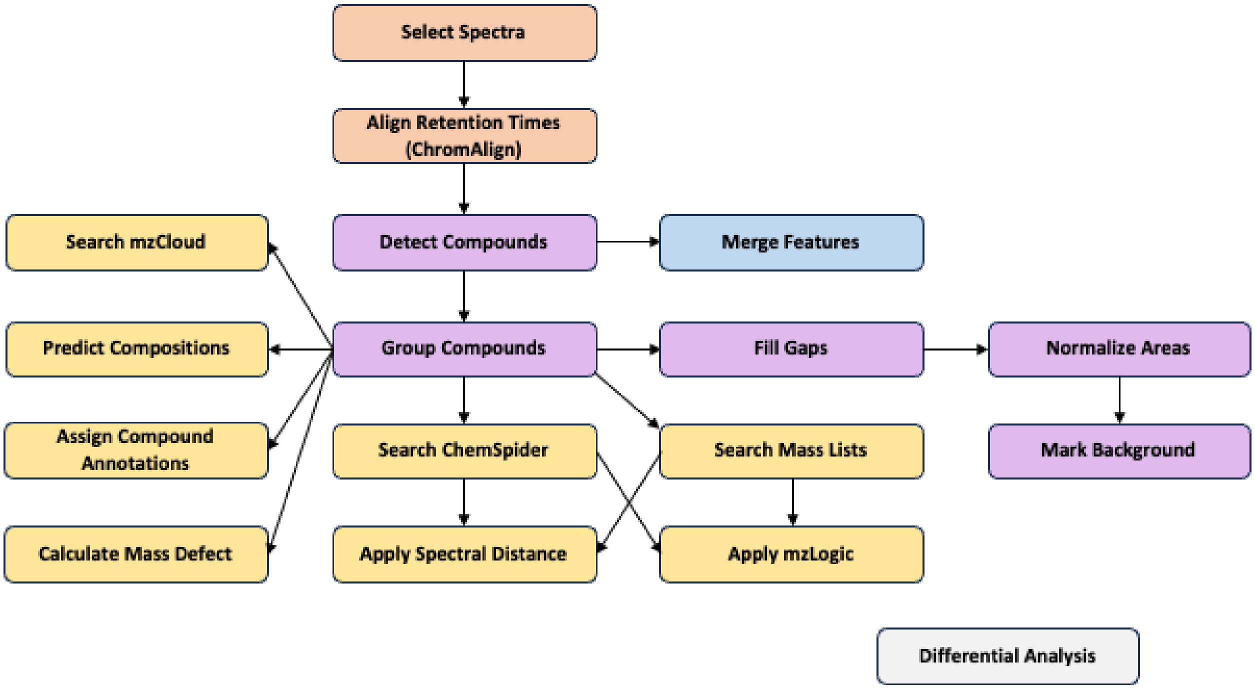

Figure S2. Workflow tree in Compound Discoverer used for analysis of all datasets. The workflow tree was adapted from the environmental workflow for unknown identifications, with modifications outlined in the following tables. The datasets were processed by date with all sites, and then by site with all dates for a total of 8 combinations.

Table S5. Parameters used for Compound Discoverer Analysis, applied in the order of the workflow as shown in **Figure S2**.

**Select Spectra**	
**1. Spectrum Properties Filter**	
Lower RT Limit	0
Upper RT Limit	13.9
**2. Scan Event Filters**	
Polarity Mode	Any
**Align Retention Times (ChromAlign)**	
**1. General Settings**	
Reference File	First sample in list
**Detect Compounds**	
**1. General Settings**	
Mass Tolerance [ppm]	5 ppm
Min. Peak Intensity	500000

Use Most Intense Isotope Only	True
**3. Peak Detection**	
Chromatographic S/N Threshold	3
Remove Baseline	False
**4. Isotope Pattern Detection**	
Group Isotopes for	Br; Cl
Ions	33/33 Checked

**Merge Features**	
**1. Peak Consolidation**	
Mass Tolerance	5 ppm
RT Tolerance [min]	0.1

**Group Compounds**	
**1. General Settings**	
Mass Tolerance	5 ppm
RT Tolerance [min]	0.1
Align Peaks	False
Preferred Ions	[M+H]+1; [M-H]-1
Area Integration	Most Common Ion
**2. Peak Rating Contributions**	
Area Contribution	3
CV Contribution	10
FWHM to Base Contribution	5
Jaggedness Contribution	5
Modality Contribution	5
Zig-Zag Index Contribution	5
**3. Peak Rating Filter**	
Peak Rating Threshold	4.5
Number of Files	3

**Fill Gaps**	
**1. General Settings**	
Mass Tolerance	5 ppm
S/N Threshold	3

**Normalize Areas**	
**1. General Settings**	
Normalization Type	Constant Median
Exclude Blanks	TRUE

**Mark Background Compounds**	
**1. General Settings**	
Max. Sample/Blank	5
Max. Blank/Sample	0
Hide Background	True

**Search mzCloud**	
**1. General Settings**	

Compound Classes	All
Library	Autoprocessesd; Reference
Search MSn Tree	False
**2. DDA Search**	
Identity Search	HighChem HighRes
Match Activation Type	True
Match Activation Energy	Match with Tolerance
Activation Energy Tolerance	20
Apply Intensity Threshold	True
Similarity Search	None
Match Factor Threshold	50
**3. DIA Search**	
Use DIA Scans for Search	True
Max. Isolation Width [Da]	500
Match Activation Type	False
Match Activation Energy	Any
Activation Energy Tolerance	100
Apply Intensity Threshold	True
Match Factor Threshold	20

**Predict Compositions**	
**1. Prediction Settings**	
Mass Tolerance	5 ppm
Min. Element Counts	C H
Max. Element Counts	C90 H190 Br3 Cl8 F18 N10 O18 P3 S5
Min. RDBE	0
Max. RDBE	40
Min. H/C	0.1
Max. H/C	3.5
Max. # Candidates	10
**2. Pattern Matching**	
Intensity Tolerance [%]	30
Intensity Threshold [%]	0.1
S/N Threshold	3
Use Dynamic Recalibration	True
**3. Fragments Matching**	
Use Fragments Matching	True
Mass Tolerance	5 ppm
S/N Threshold	3

**Assign Compound Annotations**	
**1. General Settings**	
Mass Tolerance	5 ppm
**2. Data Sources**	
Data Source #1	MassList Search
Data Source #2	Predicted Compositions

Data Source #3	mzCloud Search
Data Source #4	mzVault Search
Data Source #5	ChemSpider Search
**3. Scoring Rules**	
Use mzLogic	True
Use Spectral Distance	True
SFit Threshold	20
SFit Range	20
**4. Reprocessing**	
Clear Names	False

**Calculate Mass Defect**	
**1. Mass Defect**	
Fractional Mass	FALSE
Standard Mass Defect	FALSE
Relative Mass Defect	FALSE
Kendrick Mass Defect	TRUE
**2. Kendrick Formula**	
Formula 1	C2 F4
Formula 2	C2 F3 O
Formula 3	C2 H4
Formula 4	C3 H6
Formula 5	C8 H8

**Search ChemSpider**	
**1. Search Settings**	
Database(s)	ACToR: Aggregated Computational Toxicology Resource; DrugBank; EAWAG Biocatalysis/Biodegradation Database; EPA DSSTox; EPA Toxcast; FDA UNII - NLM
Search Mode	By Formula or Mass
Mass Tolerance	5 ppm
Max. # of results per compound	20
Max. # of Predicted Compositions to be searched per Compound	3

**Search Mass Lists**	
**1. Search Settings**	
Mass Lists	EFS HRAM Compound Database; NORMAN, UIowa, USGS
Use Retention Time	False
RT Tolerance [min]	0.5
Mass Tolerance	5 ppm

**Apply mzLogic**	
**1. Search Settings**	
Max. # Compounds	0
	10

Max. # mzCloud Similarity Results to consider per Compound	
Match Factor Threshold	30

**Apply Spectral Distance**	
**1. Pattern Matching**	
Mass Tolerance	5 ppm
Intensity Tolerance [%]	30
Intensity Threshold [%]	0.1
S/N Threshold	3
Use Dynamic Recalibration	True

**Differential Analysis**	
**1. General Settings**	
Log10 Transform Values	True
**2. Peak Rating Contributions**	
Update Peak Rating	False
Area Contribution	3
CV Contribution	10
FWHM to Base Contribution	5
Jaggedness Contribution	5
Modality Contribution	5
Zig-Zag Index	5

RRT matching + Post-Processing Filters

Table S6. 18 targeted compounds were analyzed with an inclusion list for use of relative retention time rank-order to assist with internal MassList matching. Atenolol is listed twice due to a known bimodal peak shape.

Relative Retention Order	
**RT (order of elution)**	**Compound**
1	guanylurea
2	metformin
3	atenolol (1)
4	Atenolol (2)
5	1H-benzotriazole
6	lidocaine
7	desvenlafaxine
8	thiamethoxam
9	fluconazole
10	clothianidin
11	methocarbamol
12	4/5-methyl-1H-benzotriazole
13	imidacloprid
14	sulfamethoxazole
15	bupropion
16	venlafaxine
17	citalopram
18	carbamazepine
19	fexofenadine

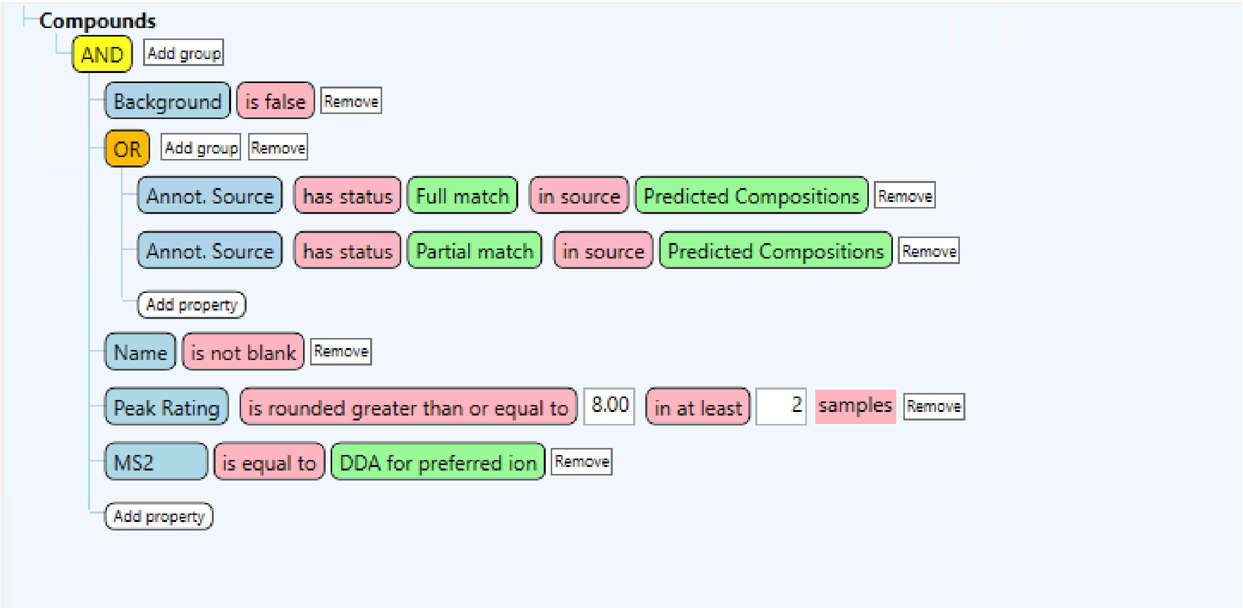

Figure S3. Suspected compound post-processing filter utilizing predicted composition matches, name match to EFS HRAM database/mzCloud/ChemSpider/NORMAN mass list, peak rating of 8+, and data dependent MS2 matches..

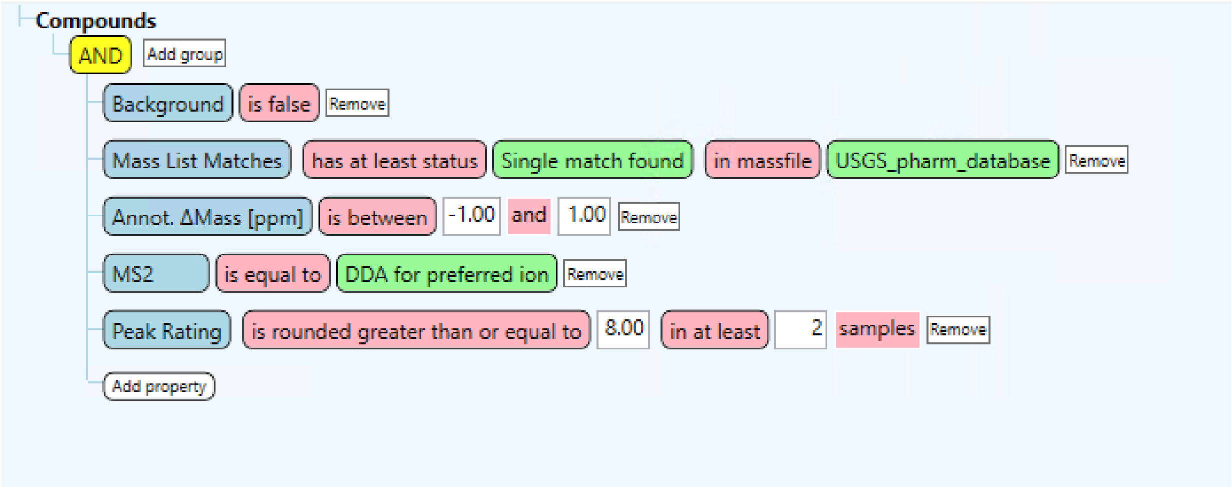

Figure S4. Suspect compound post-processing filter utilizing matches to an internal mass list including 154 compounds overlapping with the USGS targeted methods. The compounds include pharmaceuticals, pesticides, and PFAS .

Table S7. Summary of Level 2, Level 3, and Level 5 matches determined after using the workflow and parameters described above in this section.

Filters applied and # compounds				
	Upstream (combined)	Effluent (combined)	DS1 (combined)	DS2 (combined)
Level 5 - Total compounds	3010	11529	8987	6826
Level 3 - predicted compositions	642	2045	1713	1562
Level 2 - internal MassList	23	70	56	59
	January 2018	May 2018	July 2019	August 2020
Level 5 - Total compounds	17757	9134	11567	4188
Level 3 - predicted compositions	2299	1302	2114	187
Level 2 - internal MassList	34	51	80	15

Section S4: Supplemental Figures and Statistics

Retention Times

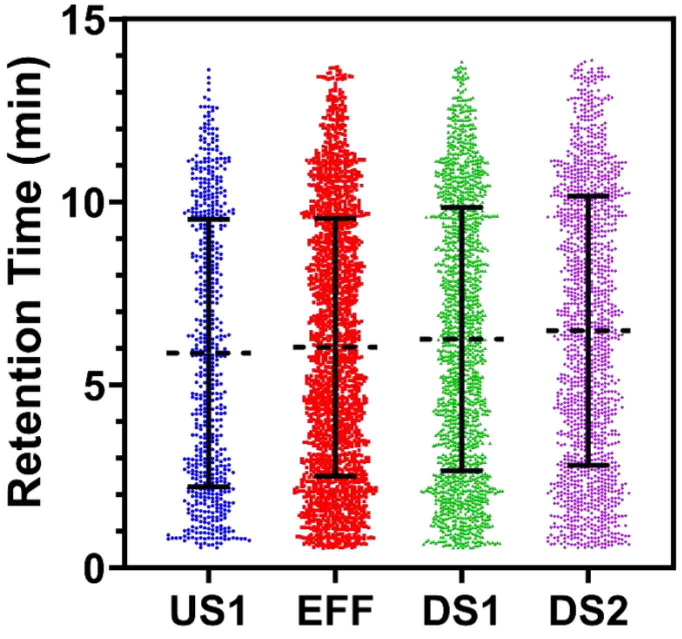

Figure S5. Retention times (RT) for Level 3 compounds listed with error bars representing means with standard deviations. There is a significant difference between means when comparing all 4 sites (ordinary 1-way ANOVA, p=0.0002). Comparing two sites at a time, the RT gradually shifts longer as the sites move downstream. Tukey's multiple comparisons were significant for 2 combinations: US1 vs DS2, p=0.0017; EFF vs DS2, p=0.0012.

Mass Distributions

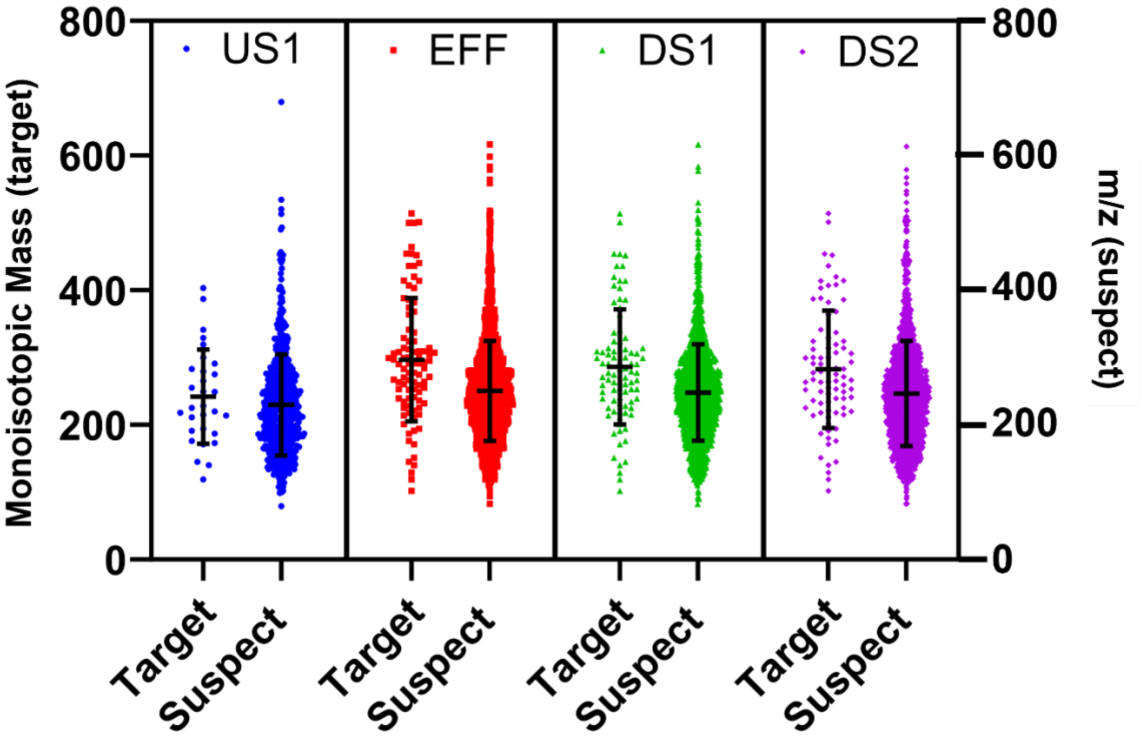

Figure S6. Dot plot distributions of masses (m/z for suspect and monoisotopic mass for target) comparing targeted and suspected contaminants. Error bars represent means with standard deviations. Targeted contaminants are Level 1 equivalent and were previously reported by the USGS[9,10] (n_{US1}=30; n_{EFF}=93; n_{DS1}=88; n_{DS2}=83). Suspect contaminants are Level 3 (n_{US1}=641; n_{EFF}=2045; n_{DS1}=1713; n_{DS2}=1562). At US1, the target and suspect contaminant distributions are not statistically significant. Nevertheless, the target and suspect contaminant distributions are statistically significant for EFF, DS1, and DS2 (Kolmogorov-Smirnov Tests: EFF $p<0.0001$; DS1 $p=0.0003$; DS2 $p=0.0035$).

All target mass distributions have mean values between 200 and 300 Da with moderate right skewness and short tails (range: 102.05 – 513.96 m/z). The shape of the target distribution is driven by the initial list of 154 possible compounds, where less extreme m/z values are possibilities. In contrast, suspect mass distributions have mean values between 200 and 300 m/z but are more heavily right skewed with longer tails (range: 79.02 – 679.51 m/z). The shapes of the suspect distributions are likely closer to the “true” distribution of organic contaminants in Muddy

Creek because the NTA method allows for a wide range of compound detections (full scan, 75 – 750 *m/z*).

Table S8. Skewness and kurtosis values for the m/z distributions listed in **Figure S6**.

Descriptive Statistics				
Site	Target		Suspect	
	Skewness	Kurtosis	Skewness	Kurtosis
US1	0.4943	-0.06854	1.229	3.037
EFF	0.4191	-0.08804	0.9249	1.704
DS1	0.4573	0.2083	0.7818	1.53
DS2	0.4563	0.04834	0.9236	1.515

Log_2 Fold Changes

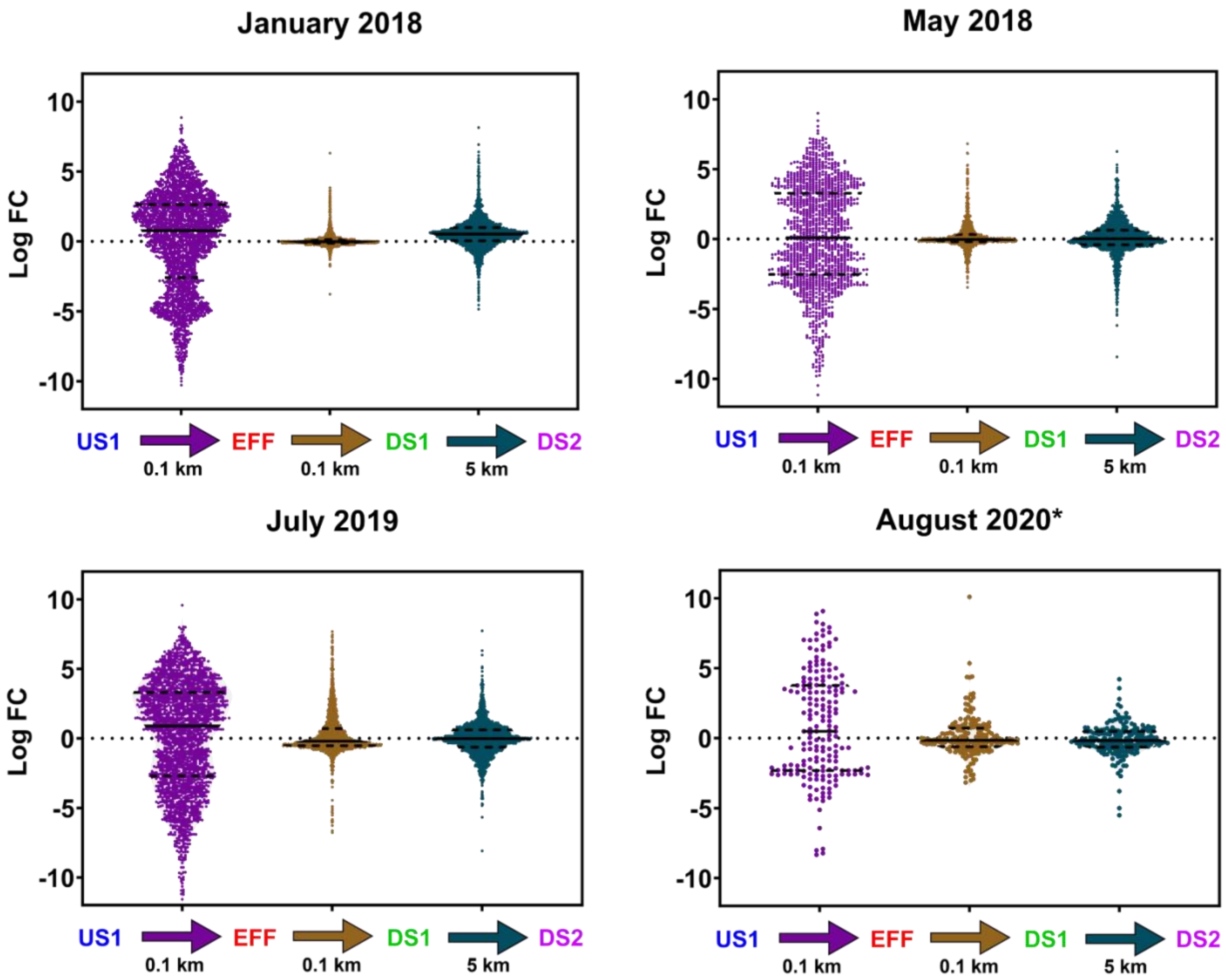

Figure S7. Log_2 fold change (Log FC) values for peak areas of individual features comparing 2 sites at a time, upstream to downstream, are shown here. Data was filtered to Level 3 prior to comparison. The purple cluster represents Log FC EFF/ Log FC US1, the brown cluster represents Log FC DS1 / Log FC EFF, and teal cluster represents Log FC DS2 / Log FC DS1. Positive values indicate upregulation and negative values indicate downregulation from the upstream site to the downstream site. Black dashes and lines represent the lower quartiles, medians, and upper quartiles. *August 2020 samples were comparing two different SPE cartridges, which ultimately lead to less suspected compounds overall.

We expect that highly positive median FC values indicate overall upregulation, meaning higher concentrations, while highly negative median FC values signify overall downregulation, reflecting lower concentrations or attenuation. As expected, peak areas increased from US1 to EFF

which mirror previous targeted results. The median FC value was modestly larger than zero (median=0.89) and exhibited high variability (sum=530.4; middle 50% range: 6 times smaller – 8 times larger). This result was expected due to the known differences in composition between the two sites, where US1 is dominated by urban and agricultural runoff and EFF is dominated by treated municipal wastewater. Next, comparing EFF and DS1 we expected a decrease in peak areas due to attenuation over the 100-meter distance downstream, in alignment with the targeted results. Instead, the change in peak areas from EFF to DS1 was only slightly negative (median=-0.20) suggesting that significant attenuation is not occurring within the 100-meters between sites. The variability from outliers was reduced (sum=532.8; middle 50% range: 0.3 times lower – 0.6 times higher) indicating that there are some compounds present in DS1 higher in concentration than at EFF, driven by a few outliers. Moving further downstream from DS1 to DS2, the fold change values of peak areas again center around 0 (median=-0.02), suggesting that the total concentrations in the complex mixture are not significantly attenuated. There is slightly more variability in peak area changes at DS2 compared to DS1 (sum=-36.5; middle 50% range: 0.5 times lower – 0.5 times higher), indicating that contaminants from the effluent may be transforming into different products and modestly attenuating.

Hierarchical Cluster Analyses

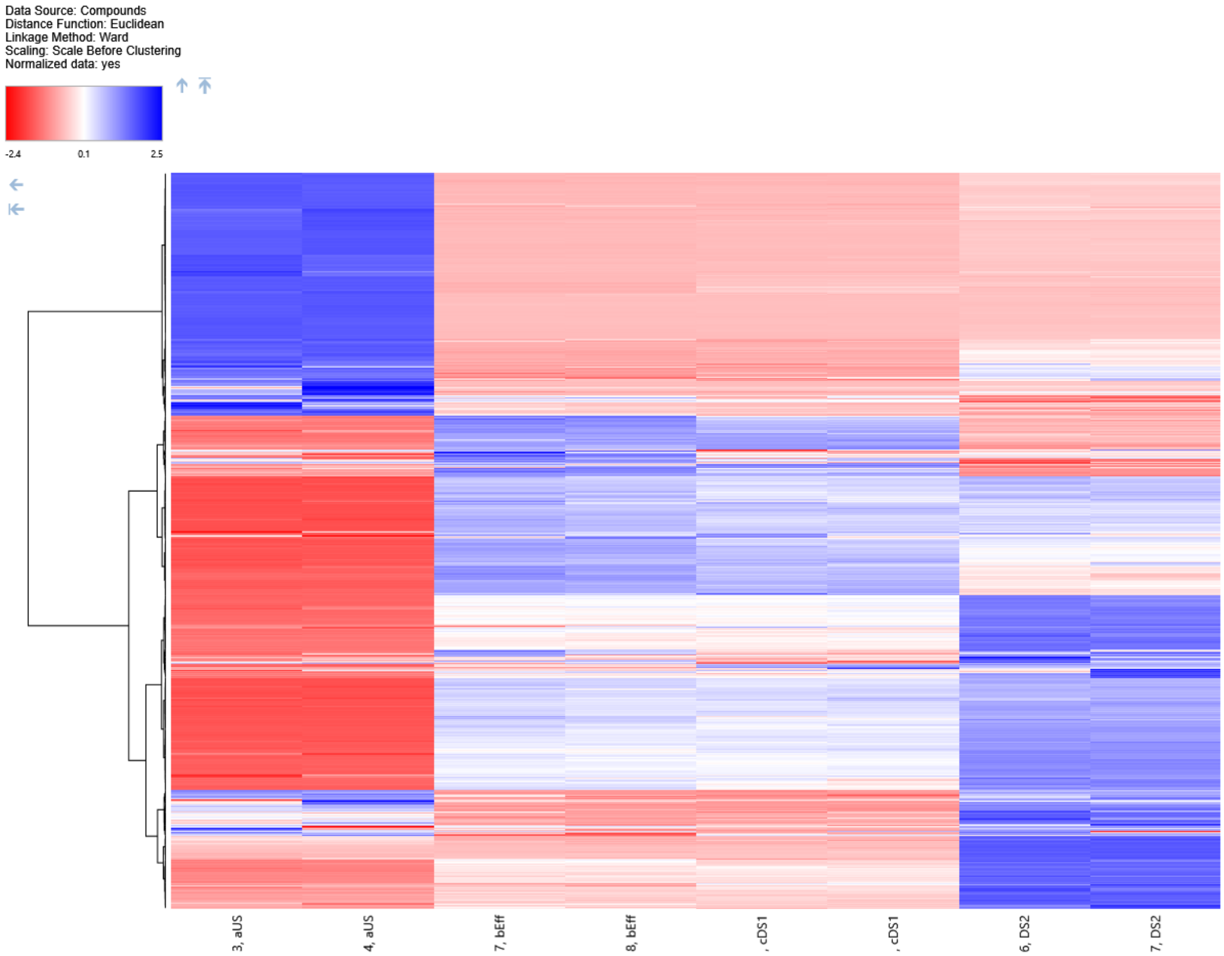

Figure S8. Hierarchal cluster analysis (HCA) of January 2018 Level 3 data (n = 2299). Columns represent sites at Muddy Creek, injected in duplicate. Horizontal lines represent individual features/compounds arranged by similarity, as represented by the dendrograms. The HCA was processed with Compound Discoverer (v3.3) using peak areas normalized to medians, Ward's linkage method, and Euclidean distancing.

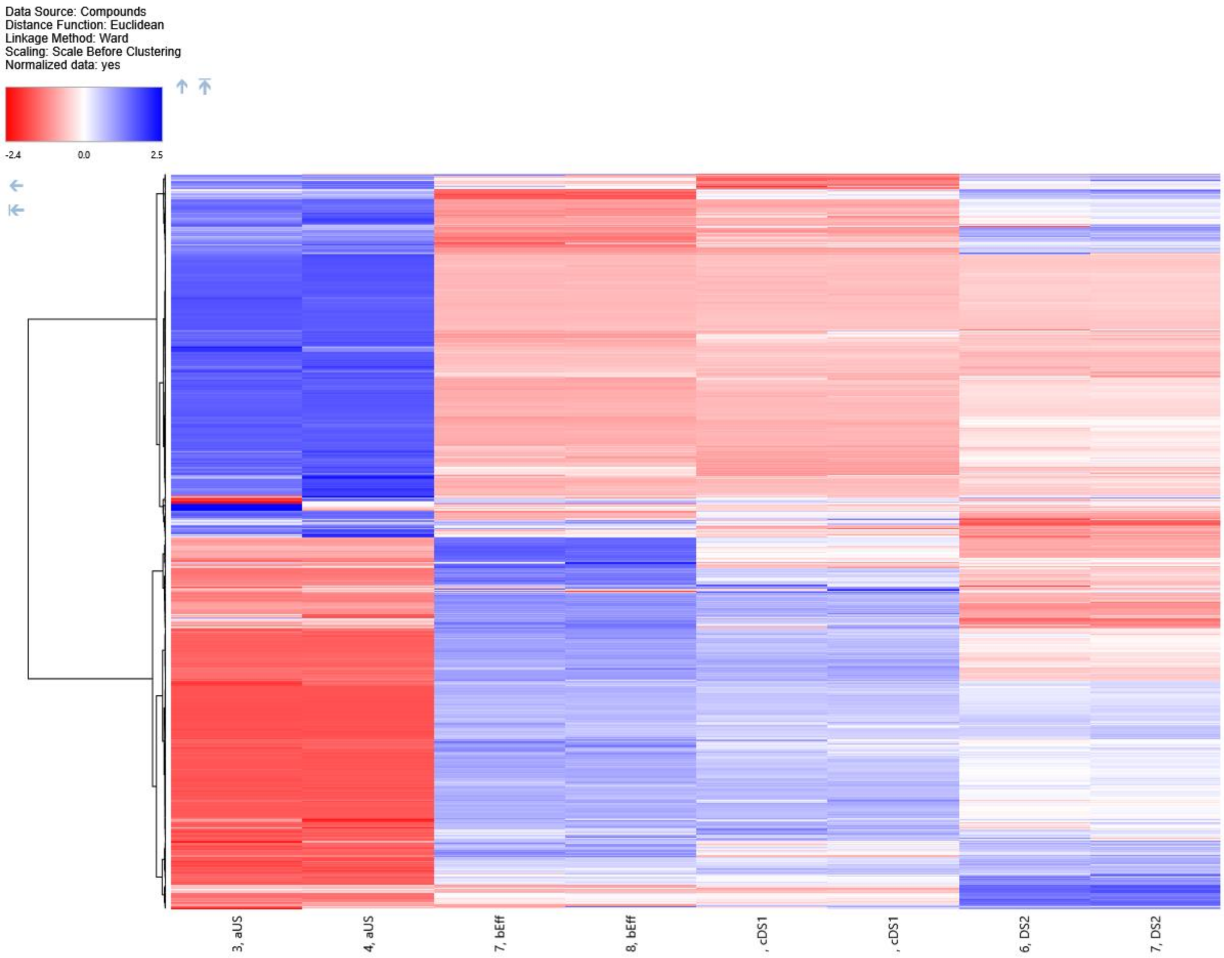

Figure S9. Hierarchal cluster analysis (HCA) of May 2018 Level 3 data (n = 1302). Columns represent sites at Muddy Creek, injected in duplicate. Horizontal lines represent individual features/compounds arranged by similarity, as represented by the dendrograms. The HCA was processed with Compound Discoverer (v3.3) using peak areas normalized to medians, Ward's linkage method, and Euclidean distancing.

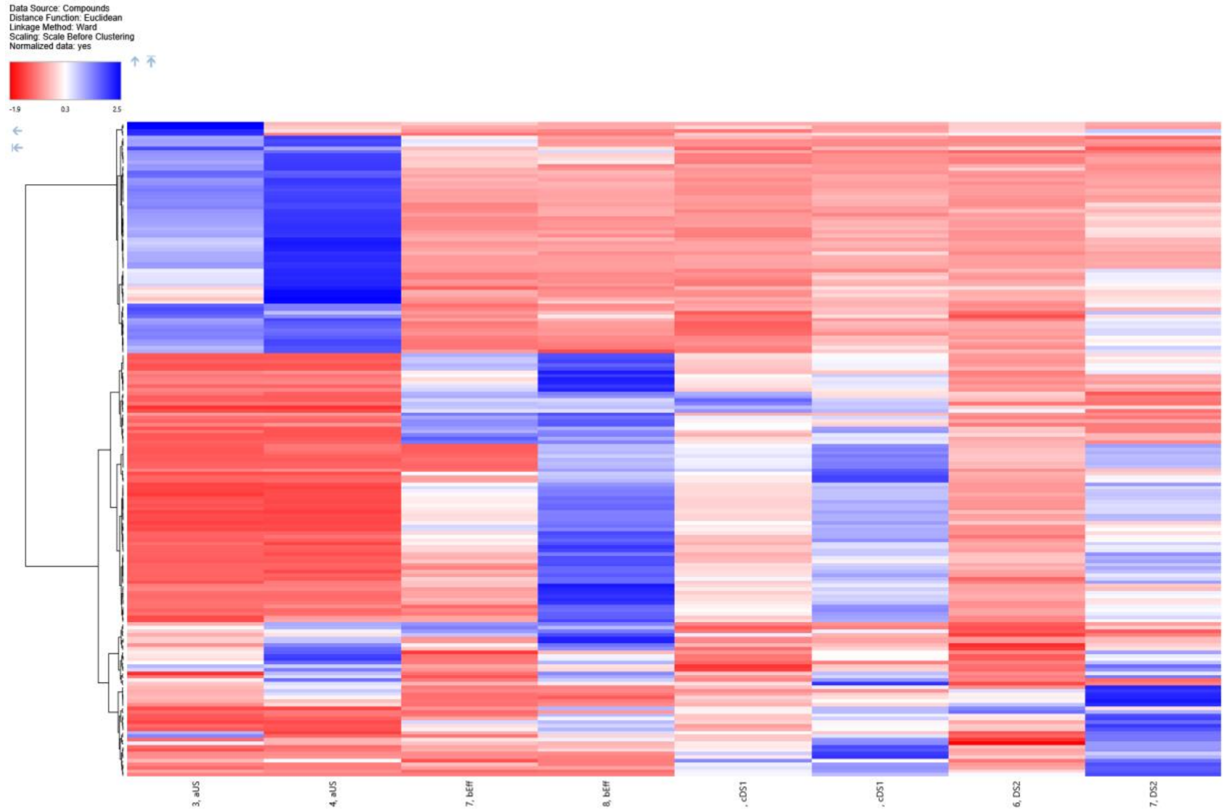

Figure S10. Hierarchal cluster analysis (HCA) of August 2020 Level 3 data (n = 187). Columns represent sites at Muddy Creek. Duplicate injections were not used in this dataset; two SPE cartridges were compared instead (Waters HLB, left columns vs. Phenomenex Strata X-CW, right columns). Waters HLB were used for all other samples, but the lack of duplicate injections greatly diminished the size of this dataset. Horizontal lines represent individual features/compounds arranged by similarity, as represented by the dendrograms. The HCA was processed with Compound Discoverer (v3.3) using peak areas normalized to medians, Ward's linkage method, and Euclidean distancing.

‘Head-to-tail’ MS2 spectra matching comparisons for named identified compounds (Figure 3 main text).

(Example suspect chemicals specifically named in the text or a figure within a given cluster that were significantly upregulated)

Cluster 1

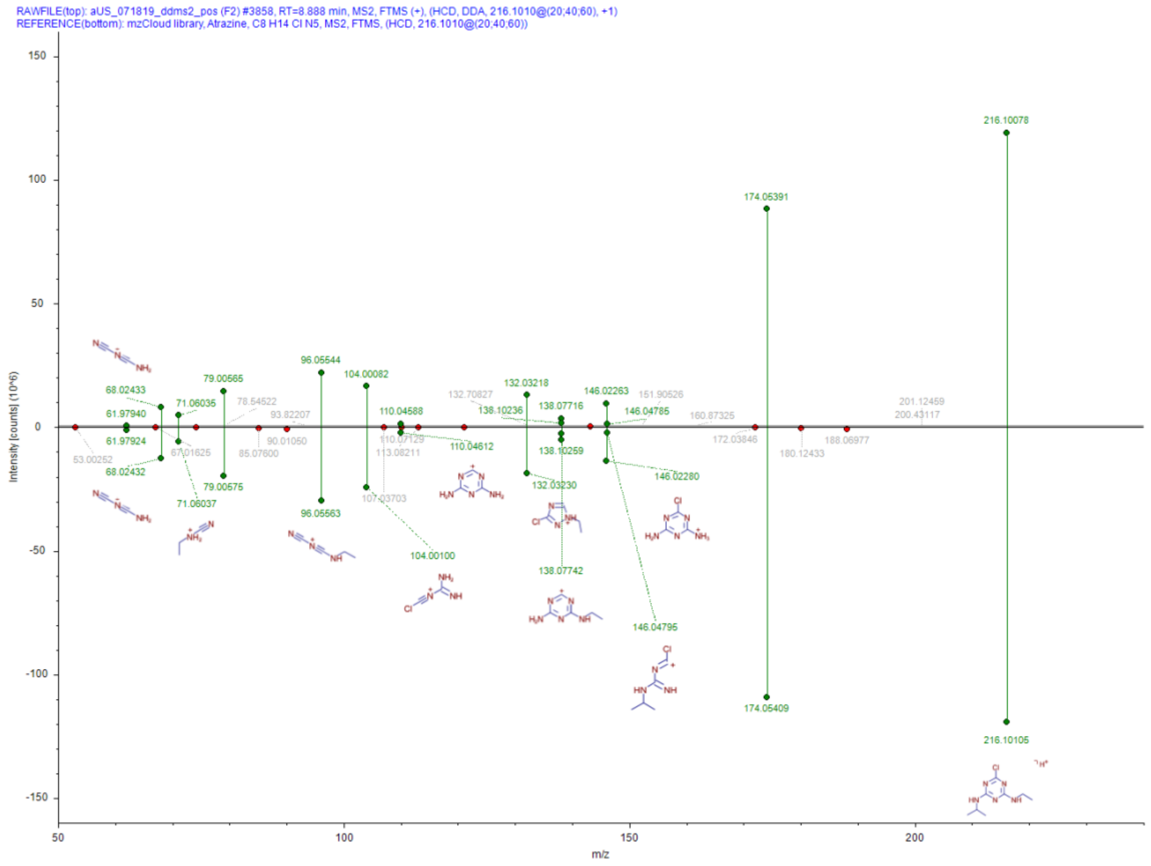

Figure S11. Atrazine detected in cluster 1 (US1). Spectra matching generated from mzCloud library match in Compound Discoverer.

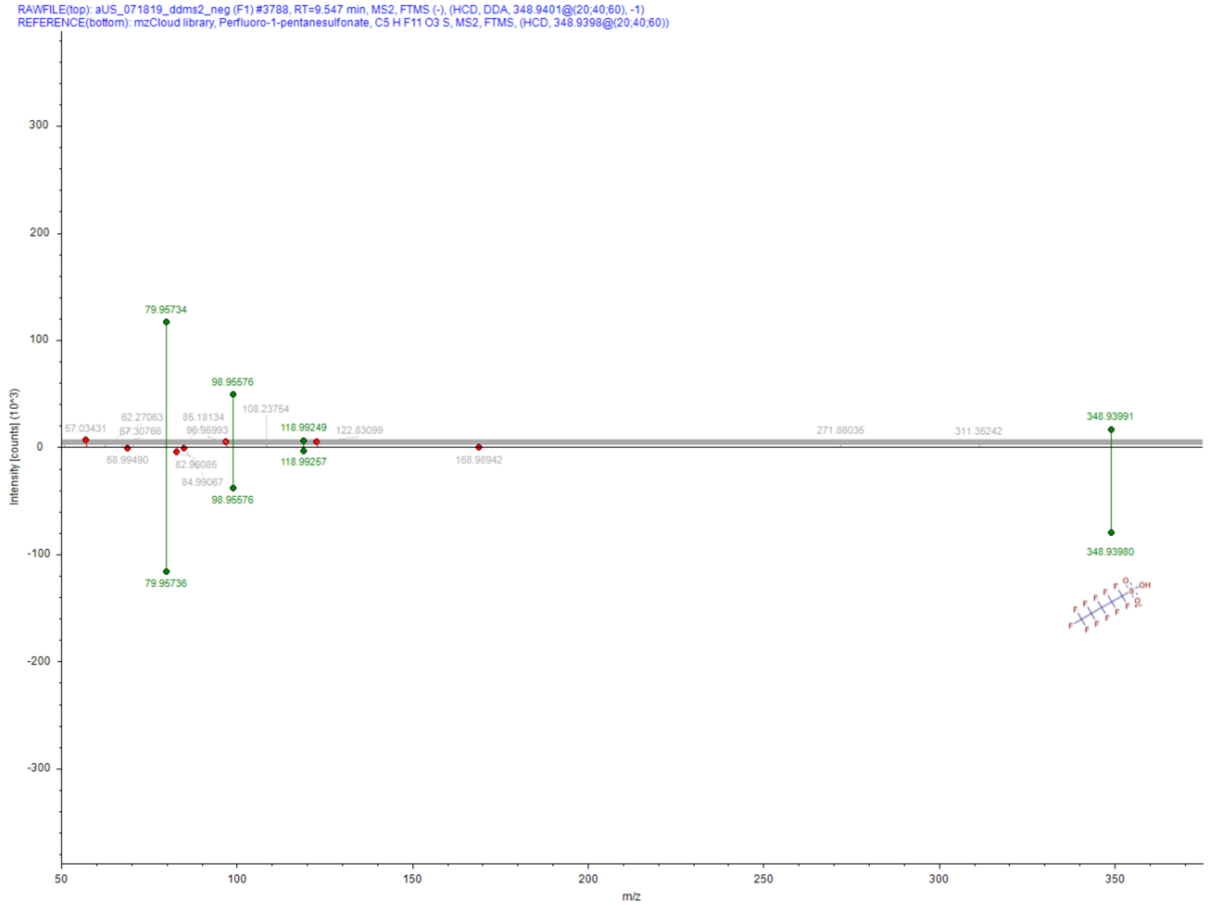

Figure S12. Perfluoropentane sulfonic acid (PFPeS) detected in cluster 1 (US1). Spectra matching generated from mzCloud library match in Compound Discoverer.

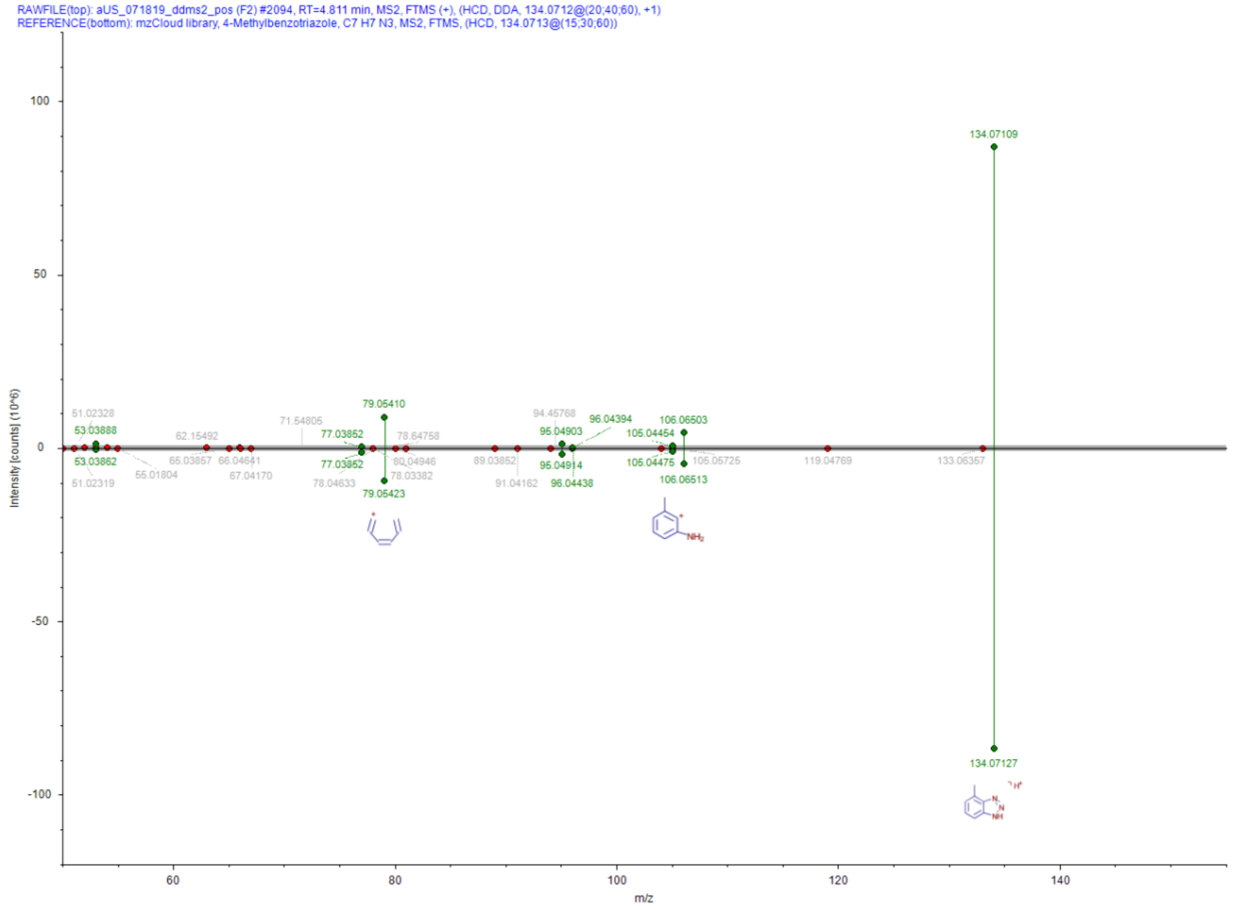

Figure S13. 4-methyl-benzotriazole detected in cluster 1 (US1). Spectra matching generated from mzCloud library match in Compound Discoverer. Based on expert knowledge, the compound could also be 5-methyl-benzotriazole, as these isomers are both common and not possible to distinguish by spectra. Often, 4- and 5-methyl benzotriazoles are found together as an industrial corrosion inhibitor mixture called ‘tolytriazole’ or referred to as ‘4-/5-methyl-1H-benzotriazole.’

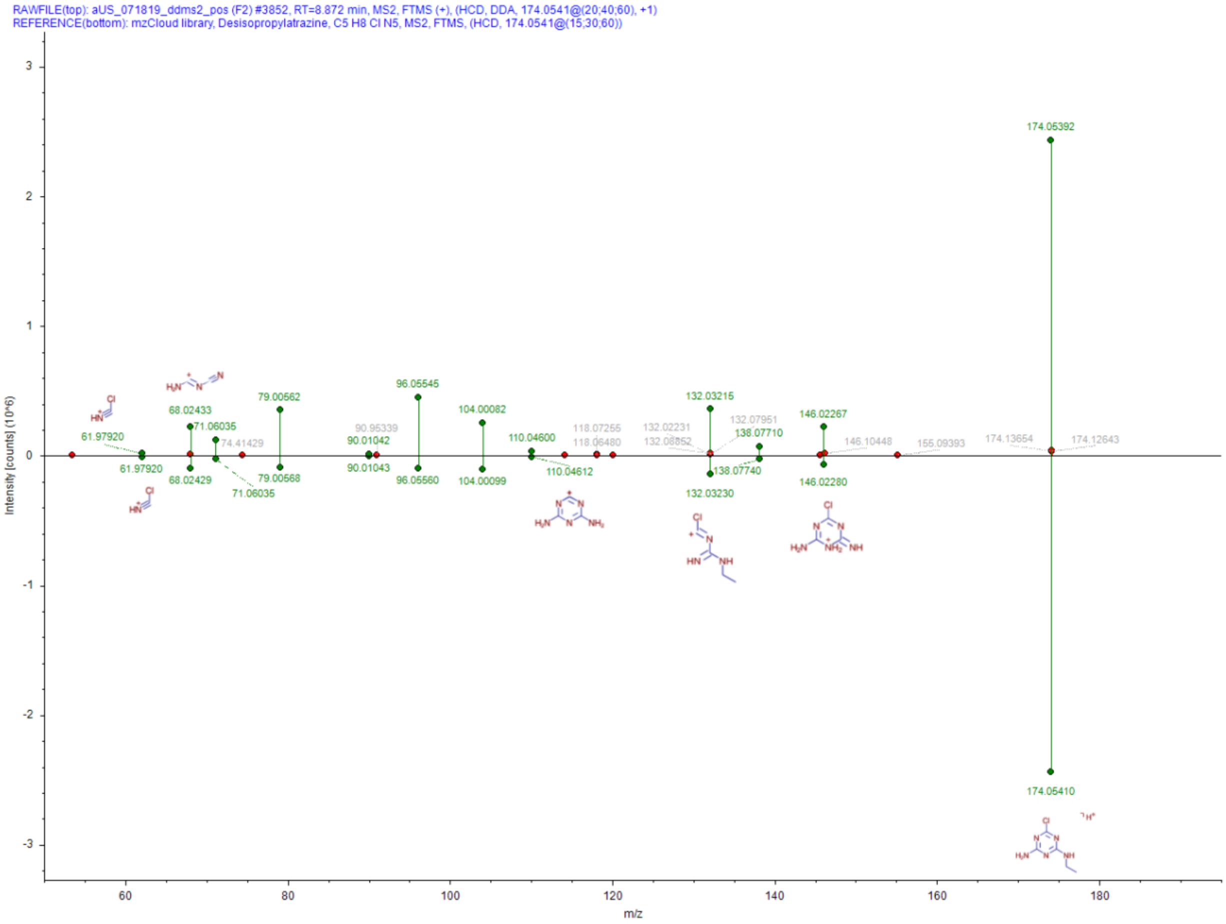

Figure S14. Desisopropylatrazine detected in cluster 1 (US1). Metabolite of atrazine. Spectra matching generated from mzCloud library match in Compound Discoverer.

Cluster 2

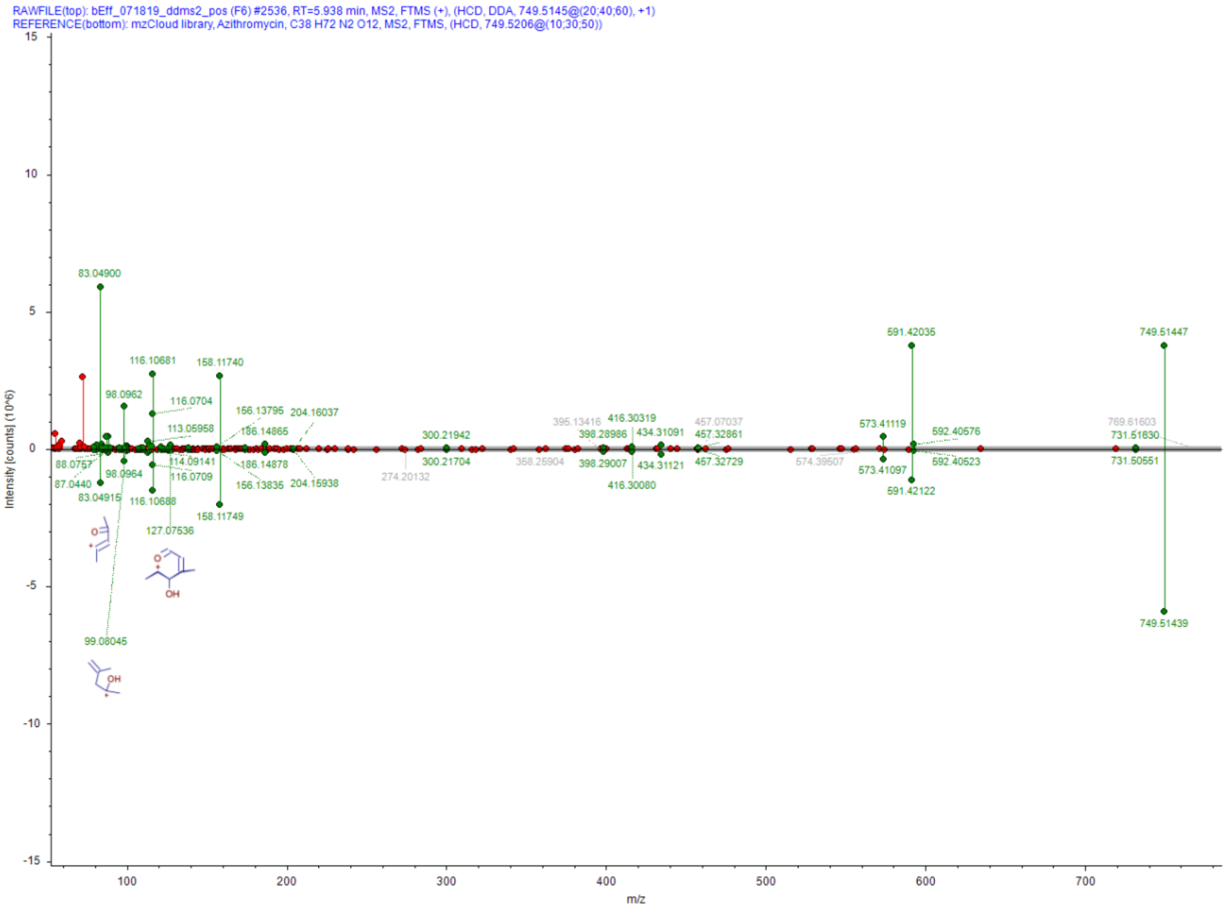

Figure S15. Azithromycin was detected in cluster 2 (EFF). Spectra matching generated from mzCloud library match in Compound Discoverer.

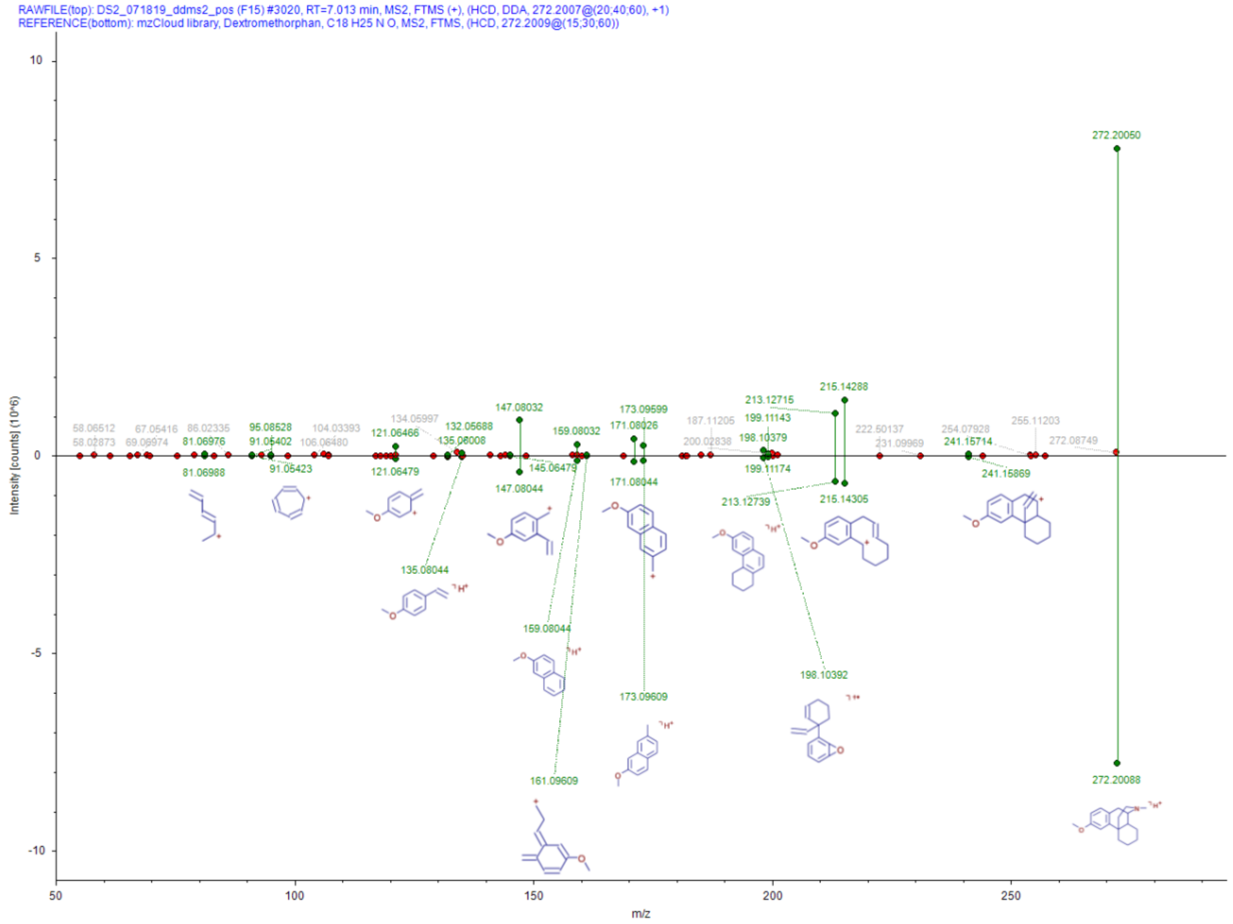

Figure S16. Dextromethorphan was detected in cluster 2 (EFF). Spectra matching generated from mzCloud library match in Compound Discoverer.

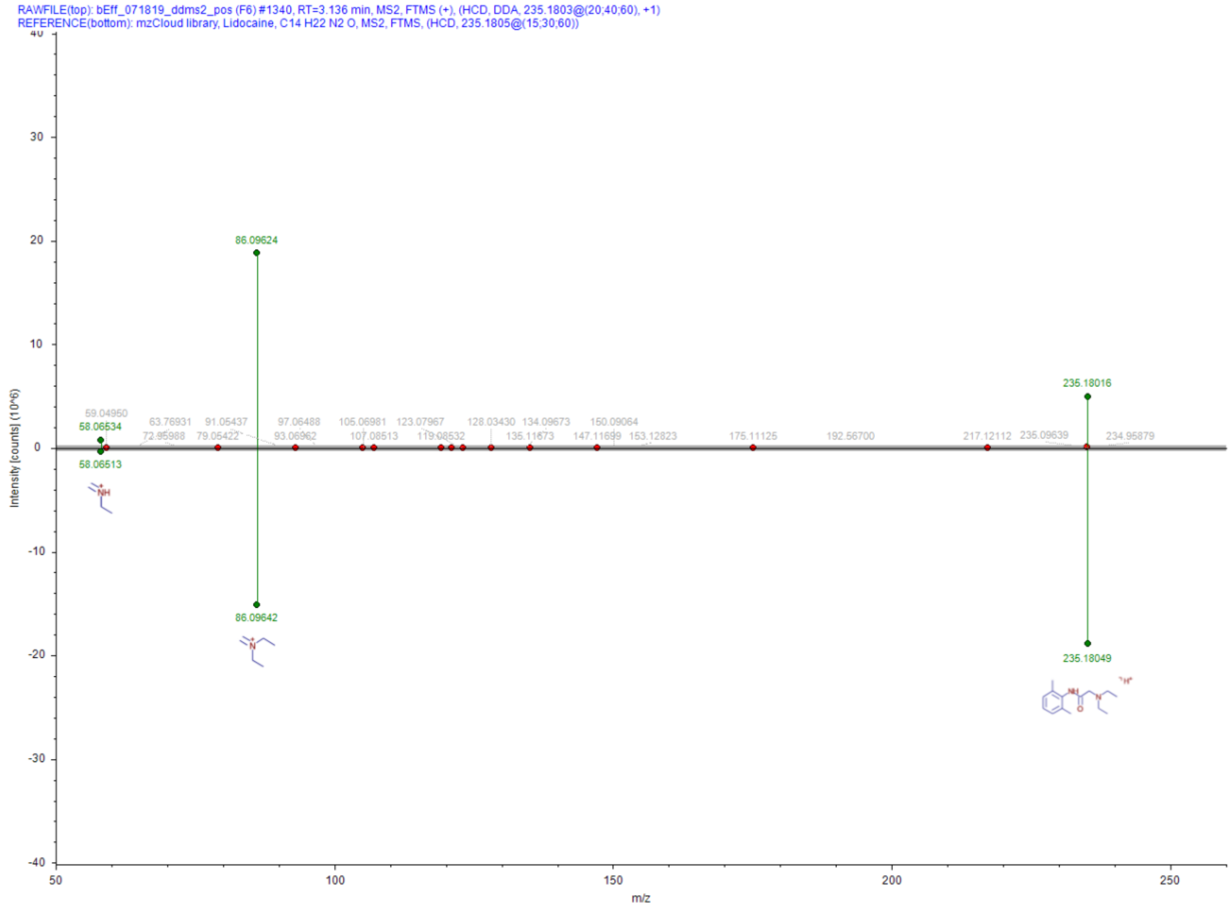

Figure S17. Lidocaine was detected in cluster 2 (EFF). Spectra matching generated from mzCloud library match in Compound Discoverer.

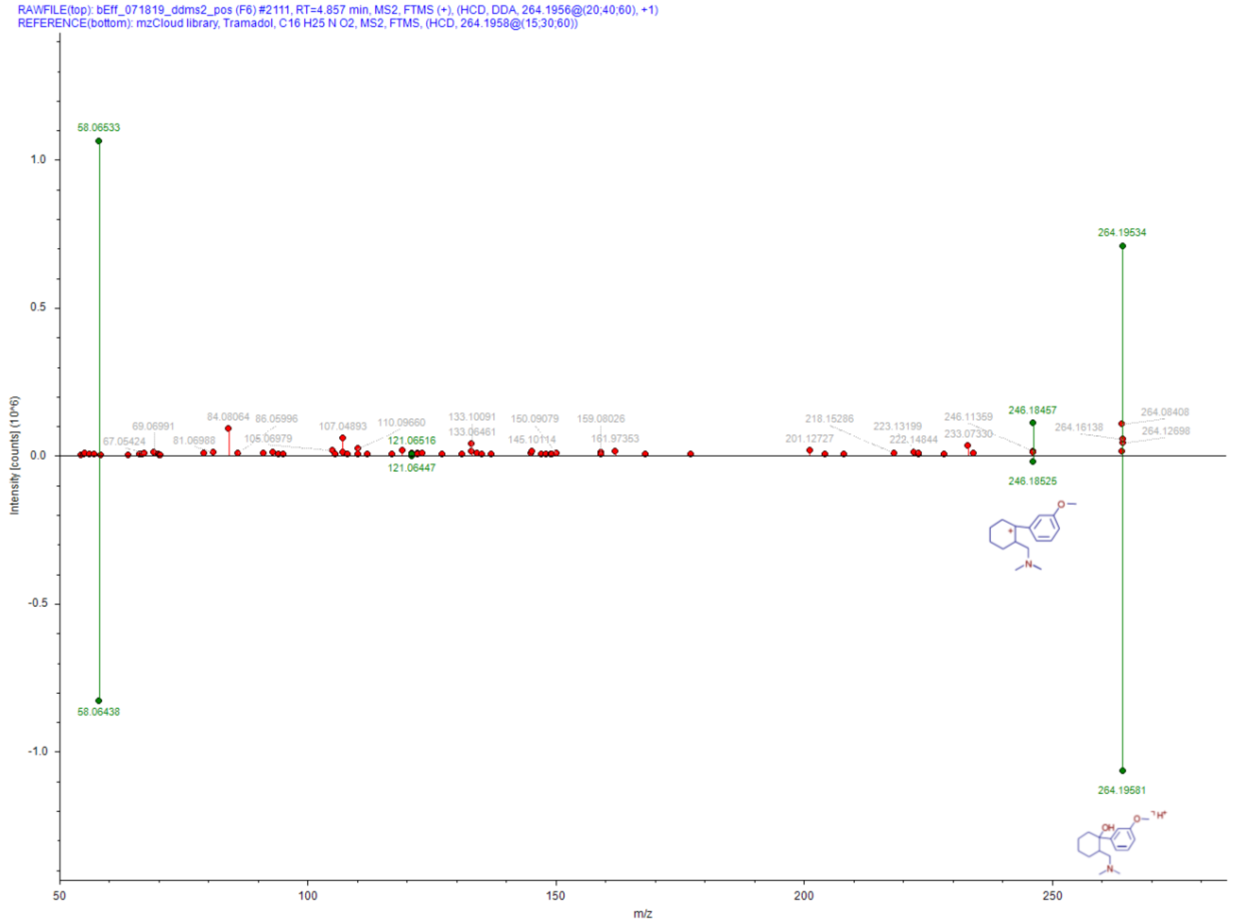

Figure S18. Tramadol was detected in cluster 2 (EFF). Spectra matching generated from mzCloud library match in Compound Discoverer.

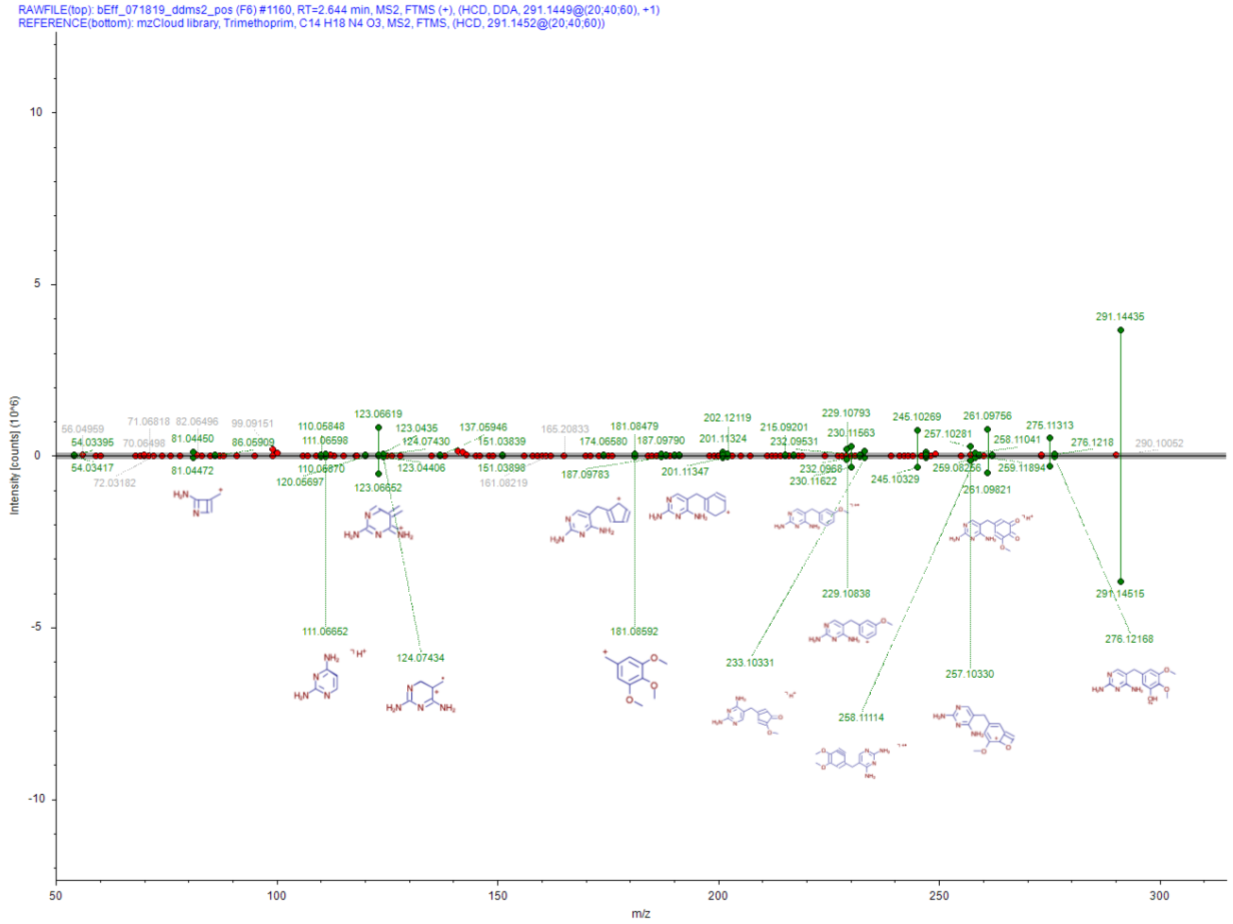

Figure S19. Trimethoprim was detected in cluster 2 (EFF). Spectra matching generated from mzCloud library match in Compound Discoverer.

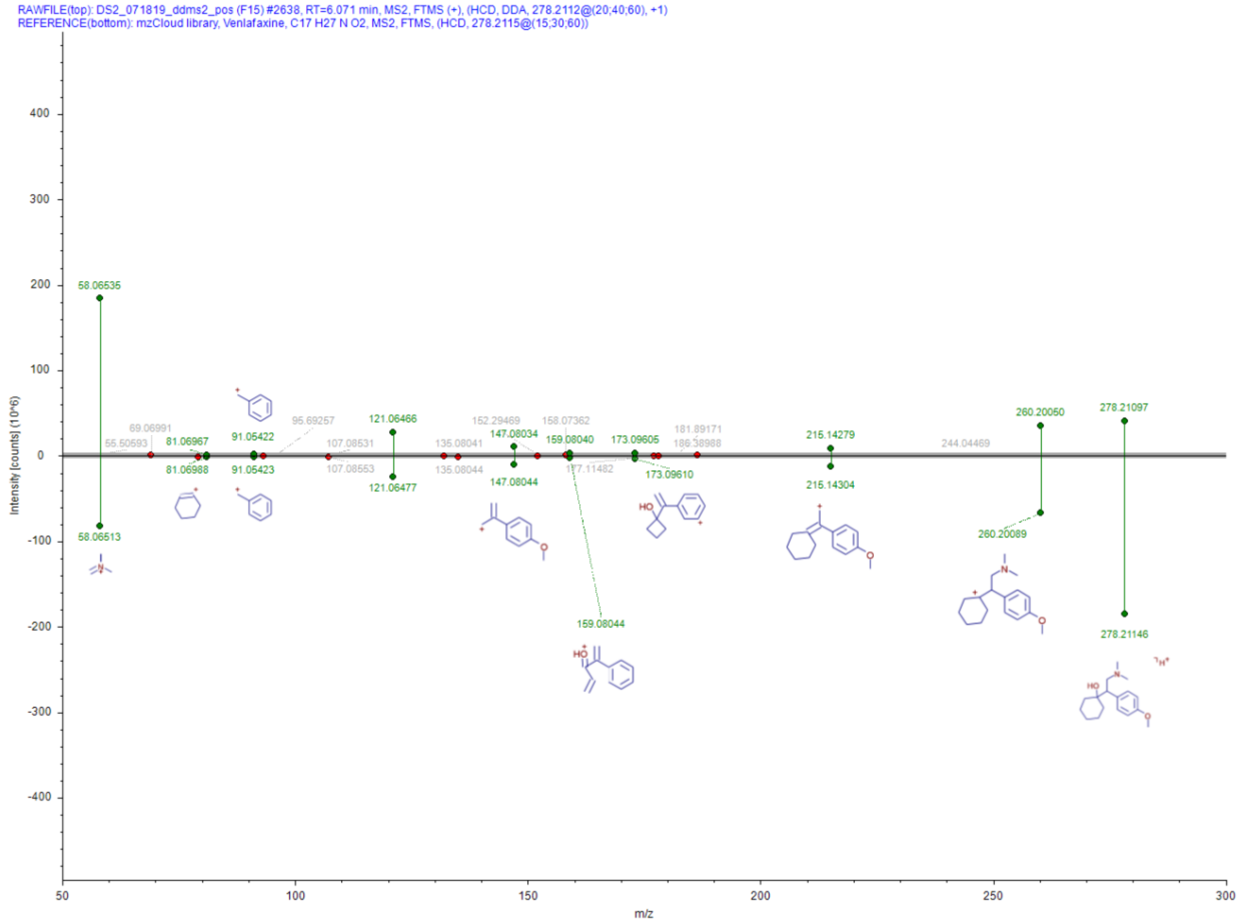

Figure S20. Venlafaxine was detected in cluster 2 (EFF). Spectra matching generated from mzCloud library match in Compound Discoverer.

Cluster 3

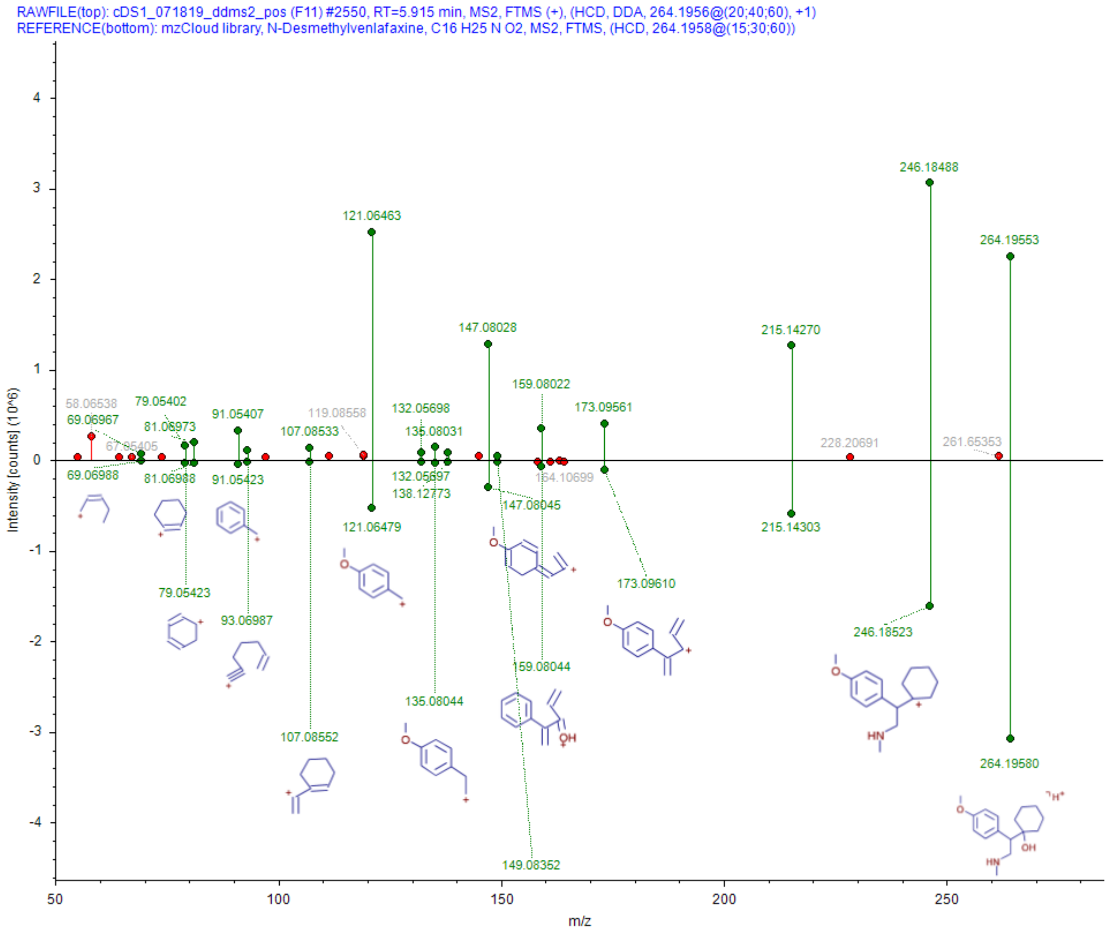

Figure S21. N-desmethylvenlafaxine detected in cluster 3 (DS1). Metabolite of venlafaxine. Spectra matching generated from mzCloud library match in Compound Discoverer.

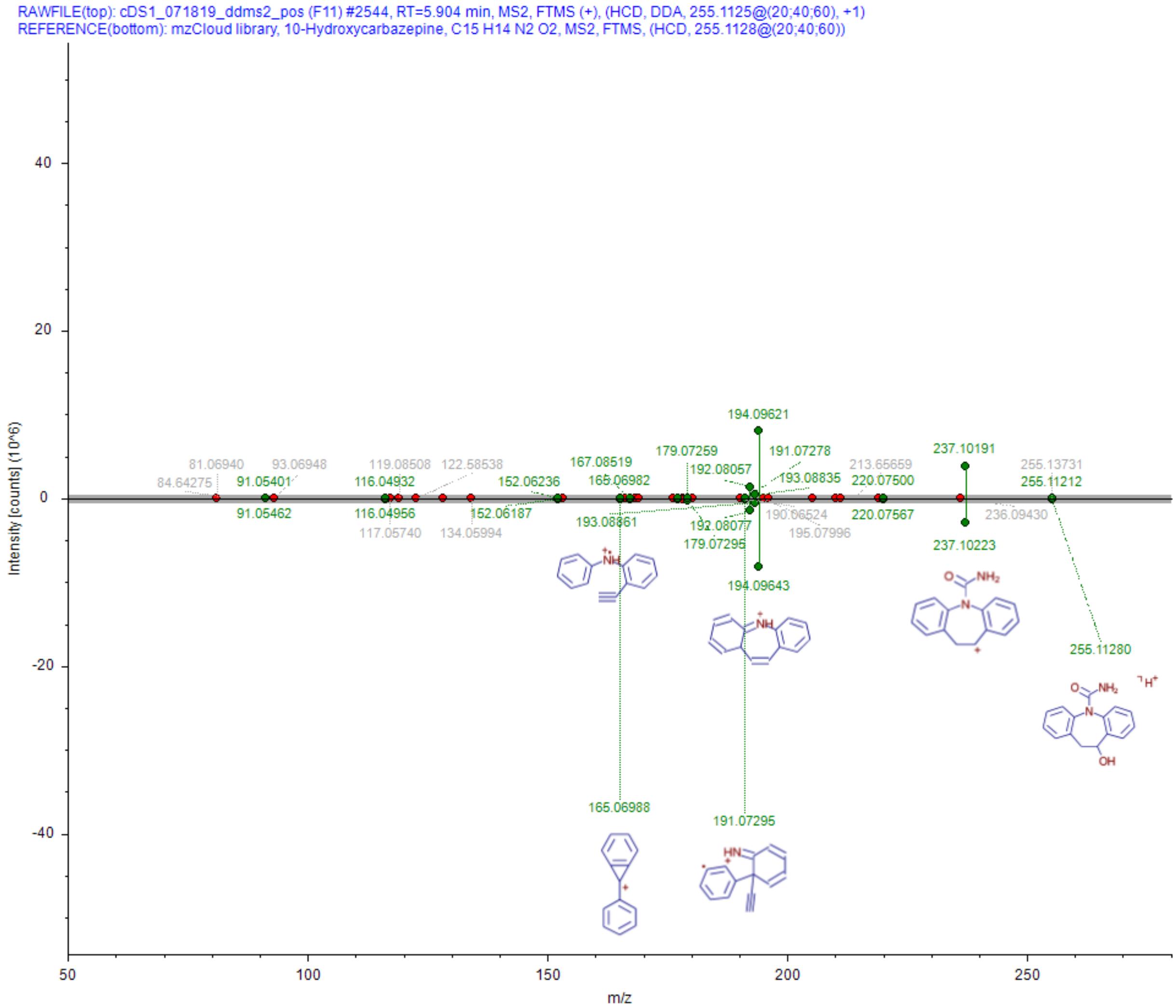

Figure S22. 10-hydroxycarbazepine detected in cluster 3 (DS1). Metabolite of carbamazepine. Spectra matching generated from mzCloud library match in Compound Discoverer.

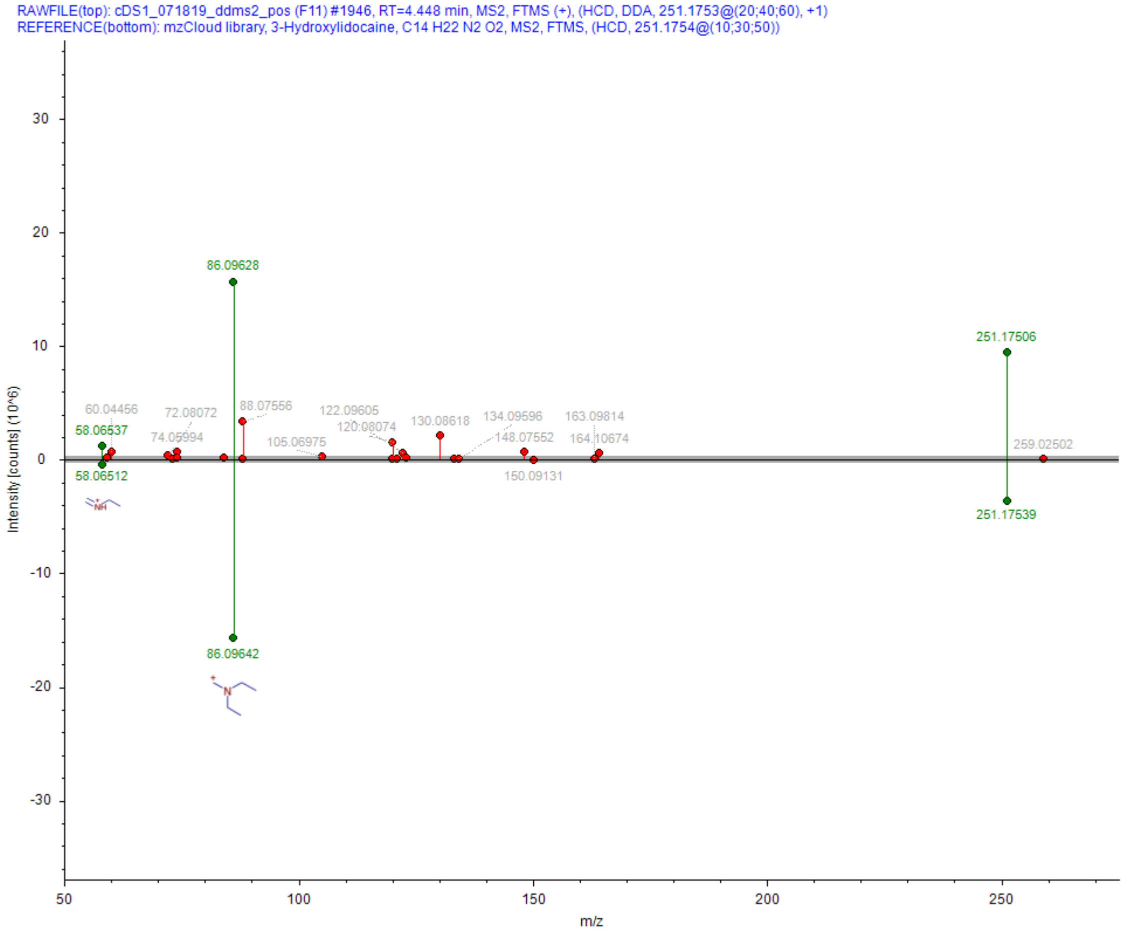

Figure S23. 3-hydroxylidocaine detected in cluster 3 (DS1). Metabolite of lidocaine. Spectra matching generated from mzCloud library match in Compound Discoverer.

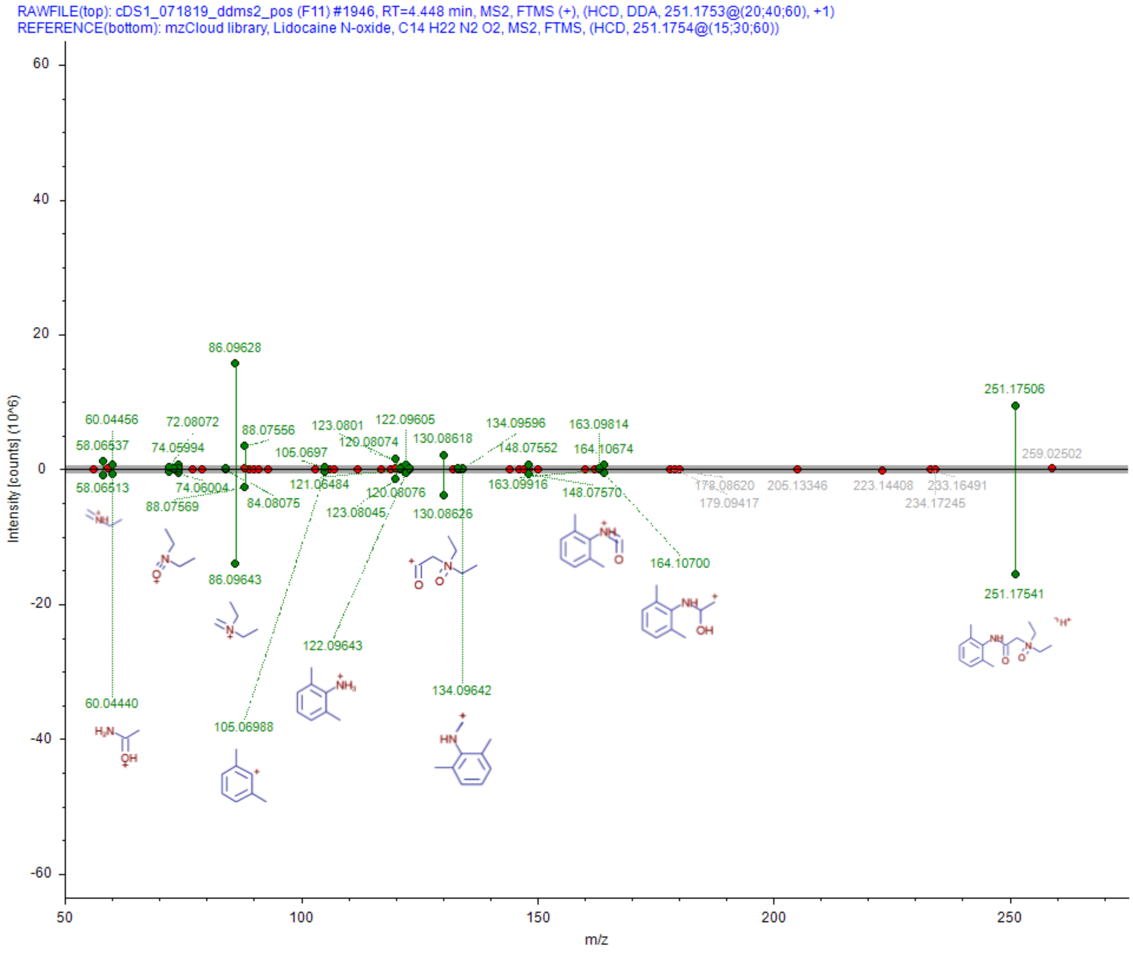

Figure S24. Lidocaine N-oxide detected in cluster 3 (DS1). Metabolite of lidocaine. Spectra matching generated from mzCloud library match in Compound Discoverer.

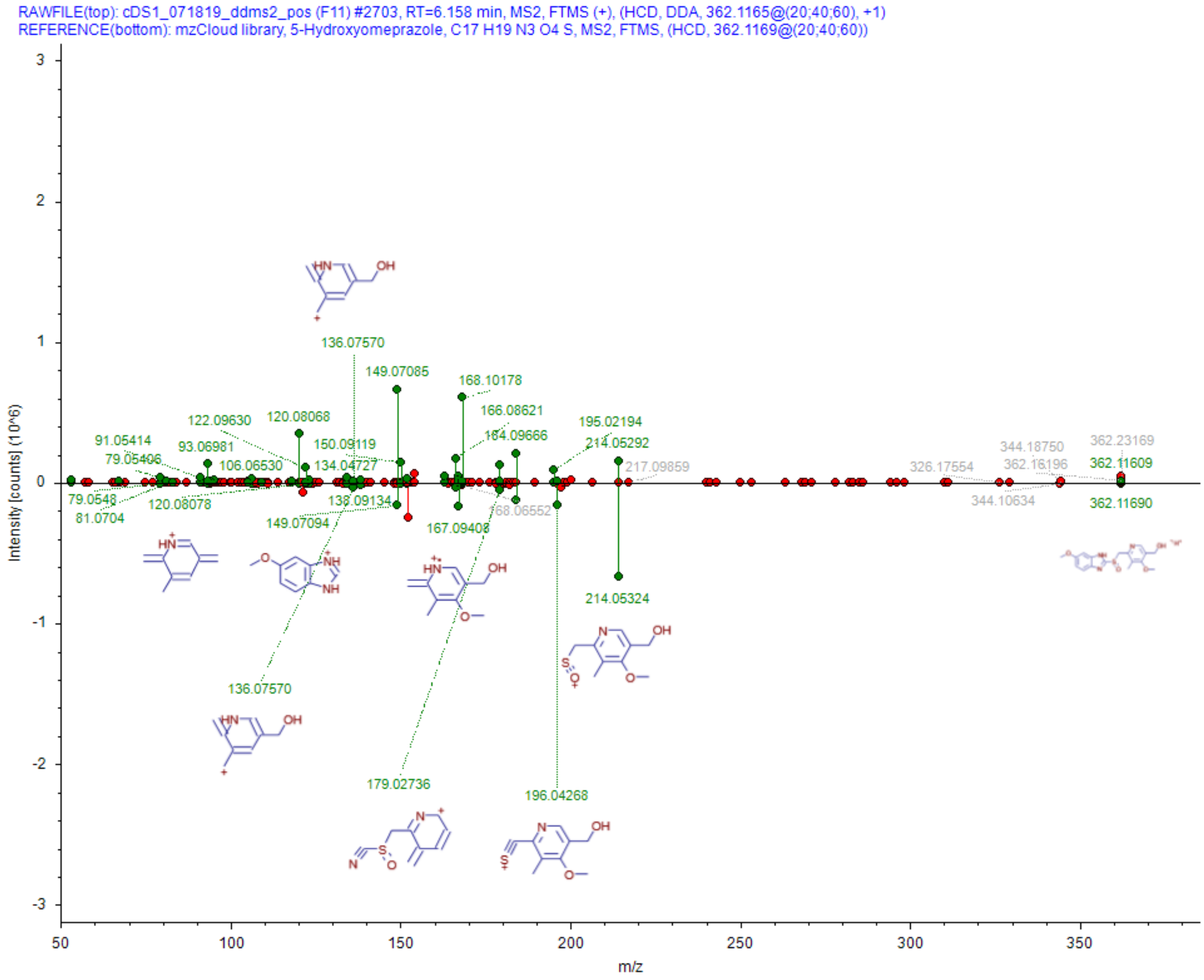

Figure S25. 5-hydroxyomeprazole detected in cluster 3 (DS1). Metabolite of omeprazole. Spectra matching generated from mzCloud library match in Compound Discoverer.

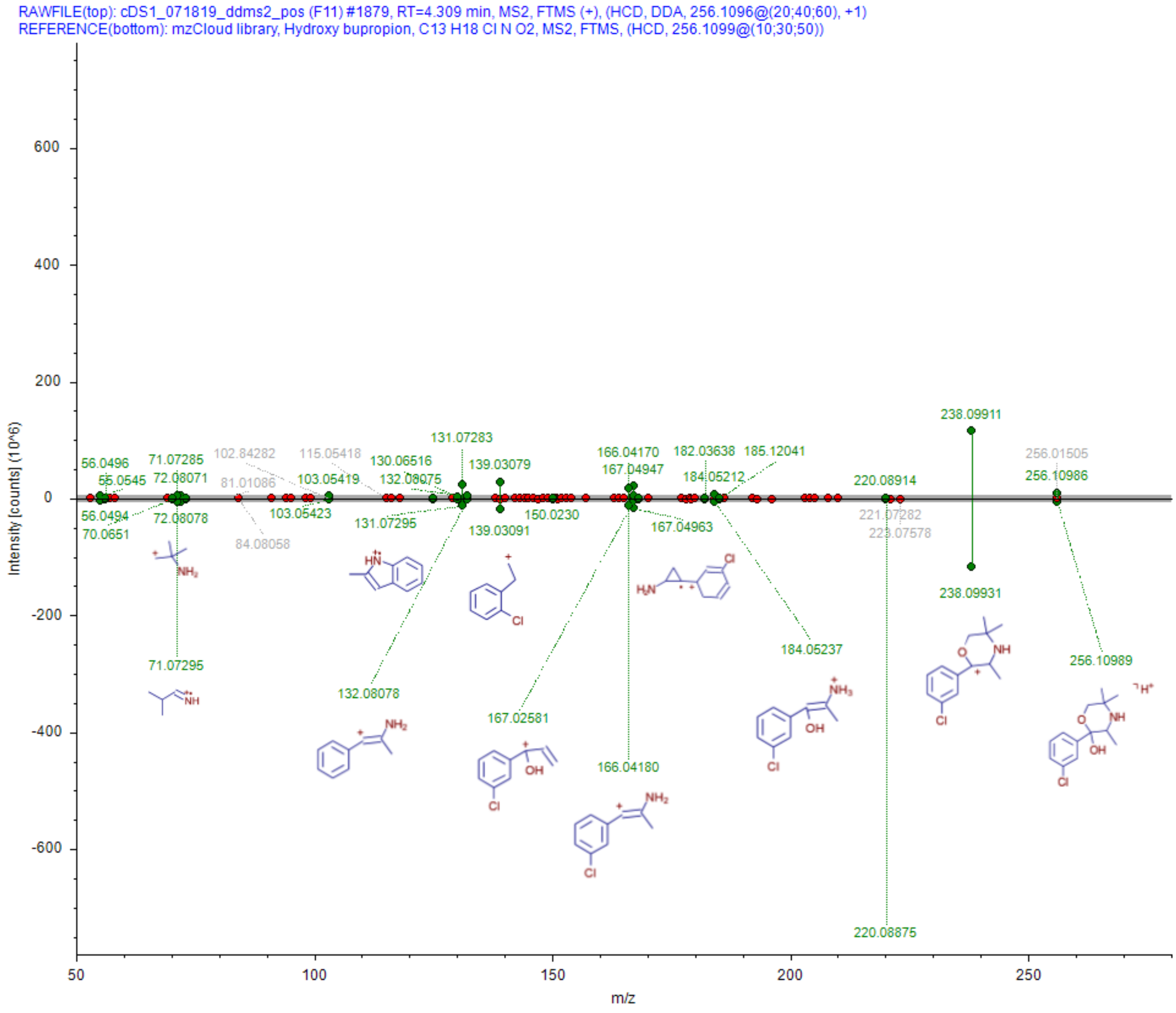

Figure S26. Hydroxybupropion detected in cluster 3 (DS1). Metabolite of bupropion. Spectra matching generated from mzCloud library match in Compound Discoverer.

Cluster 4

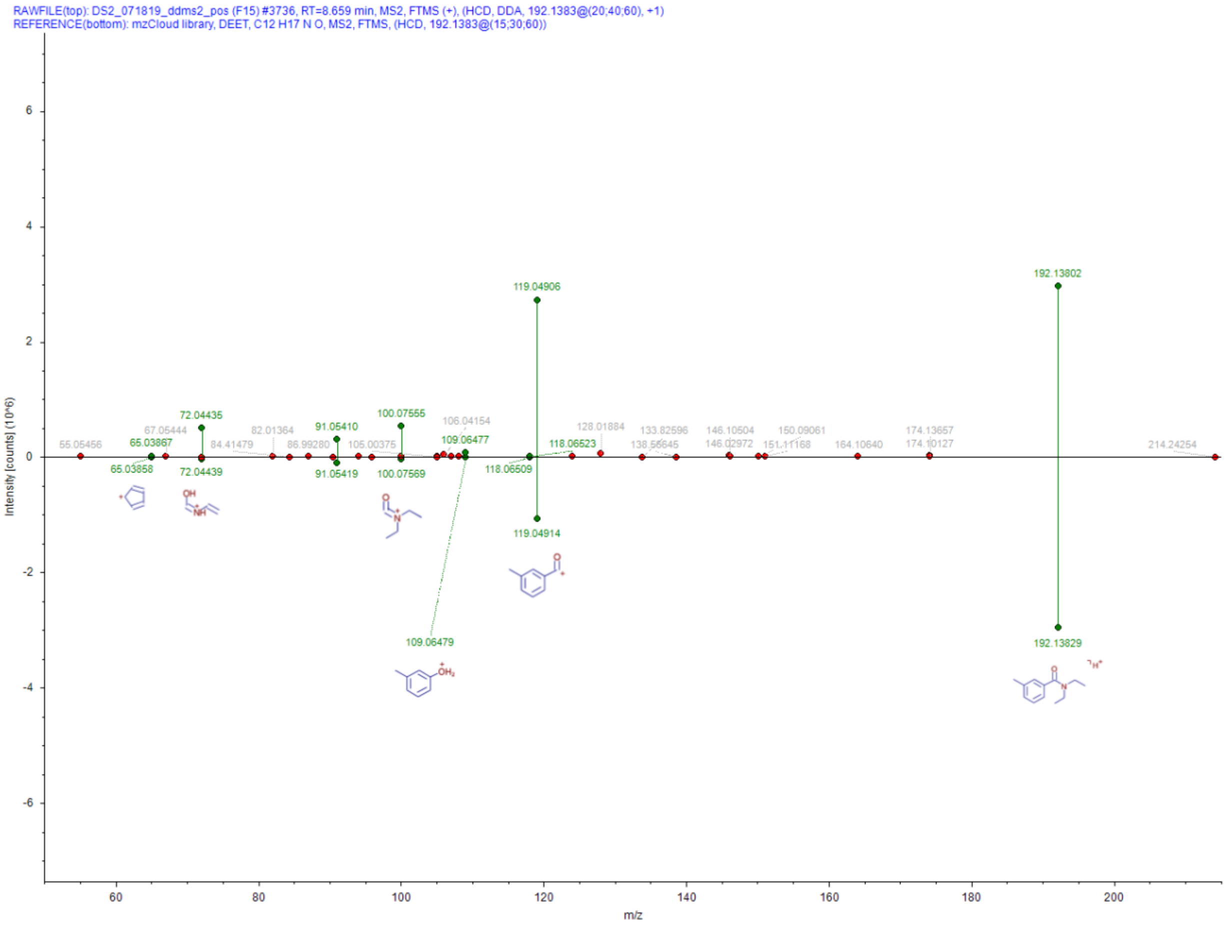

Figure S27. DEET was detected in cluster 4 (DS2). Spectra matching generated from mzCloud library match in Compound Discoverer.

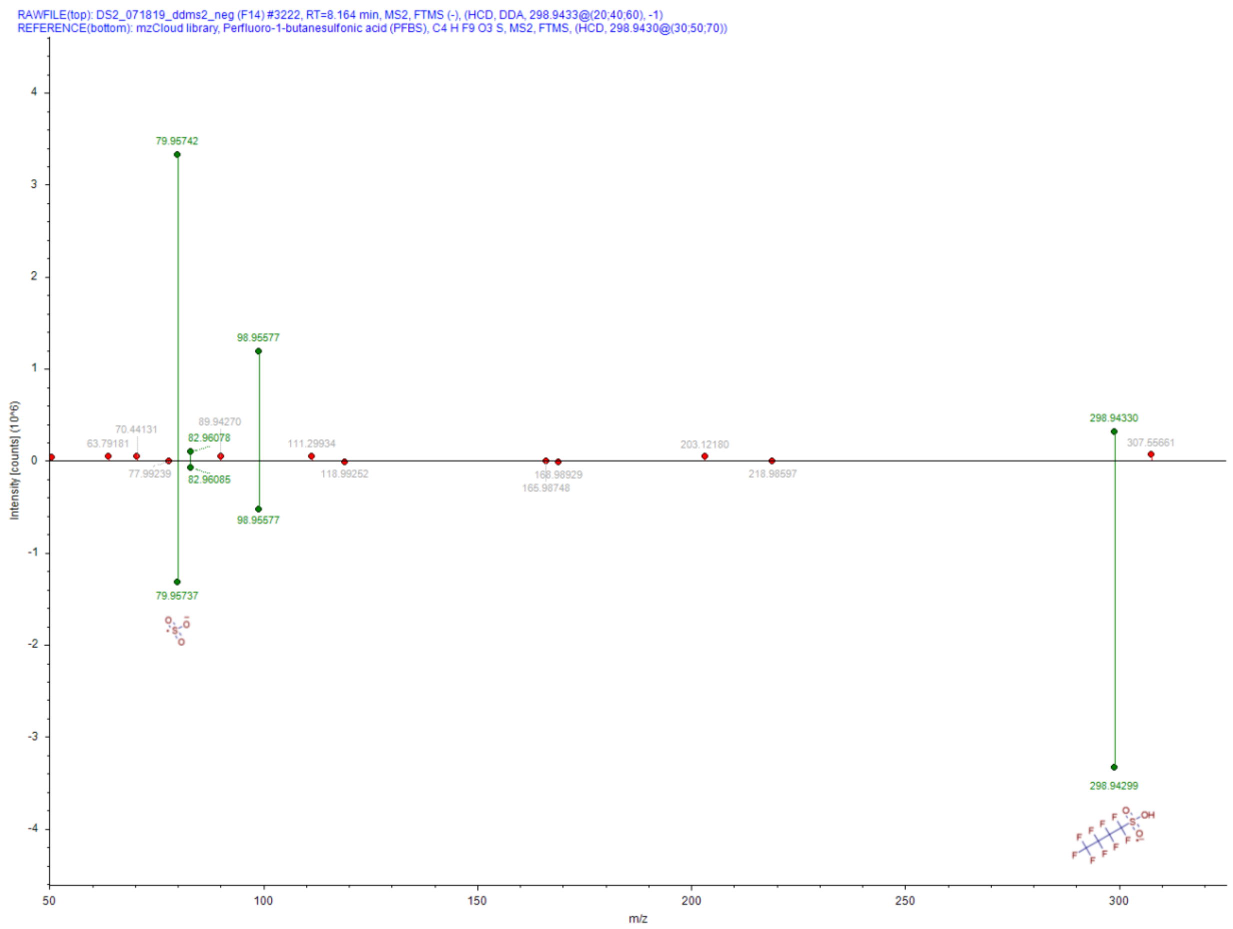

Figure S28. Perfluorobutanesulfonic acid (PFBS) was detected in cluster 4 (DS2). Spectra matching generated from mzCloud library match in Compound Discoverer.

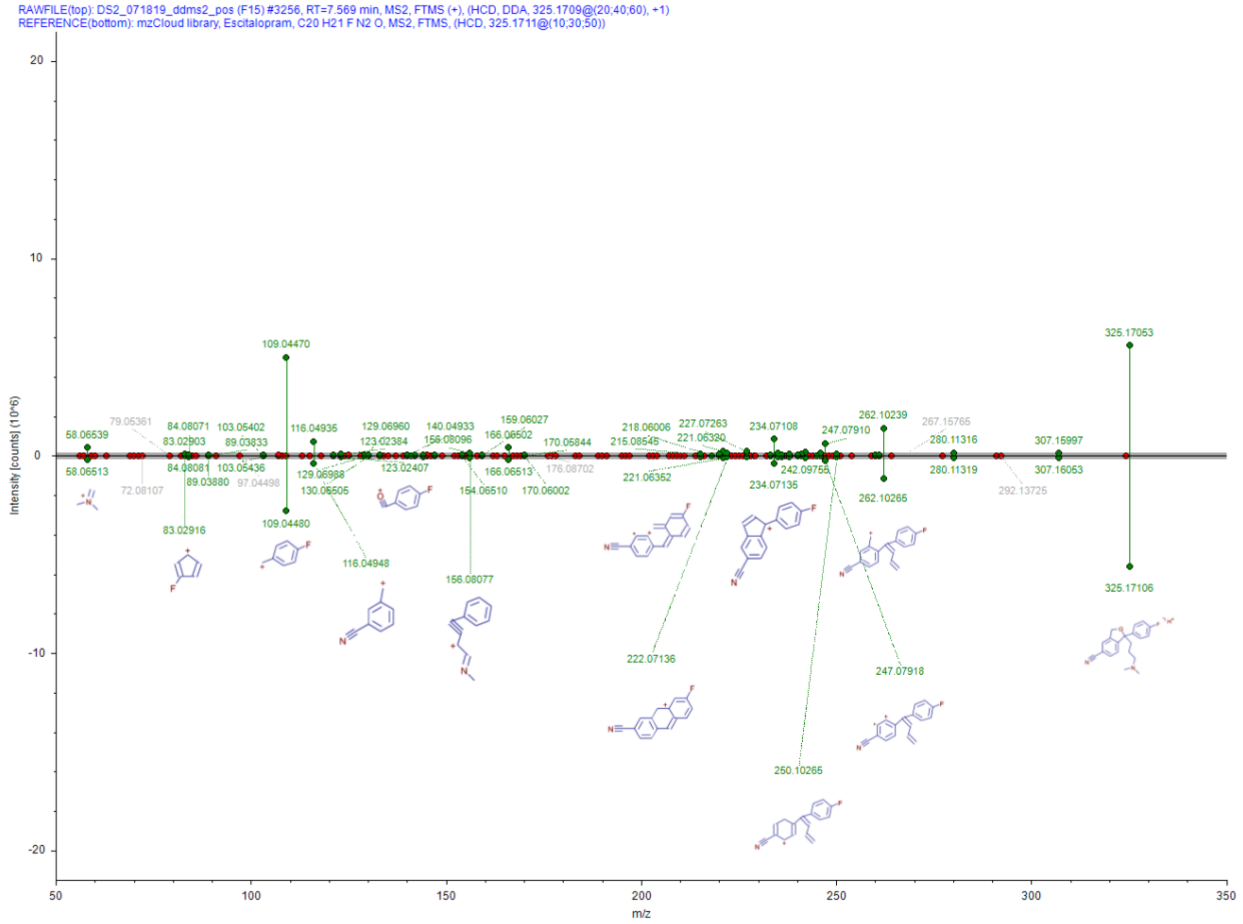

Figure S29. Escitalopram was detected in cluster 4 (DS2). Spectra matching generated from mzCloud library match in Compound Discoverer.

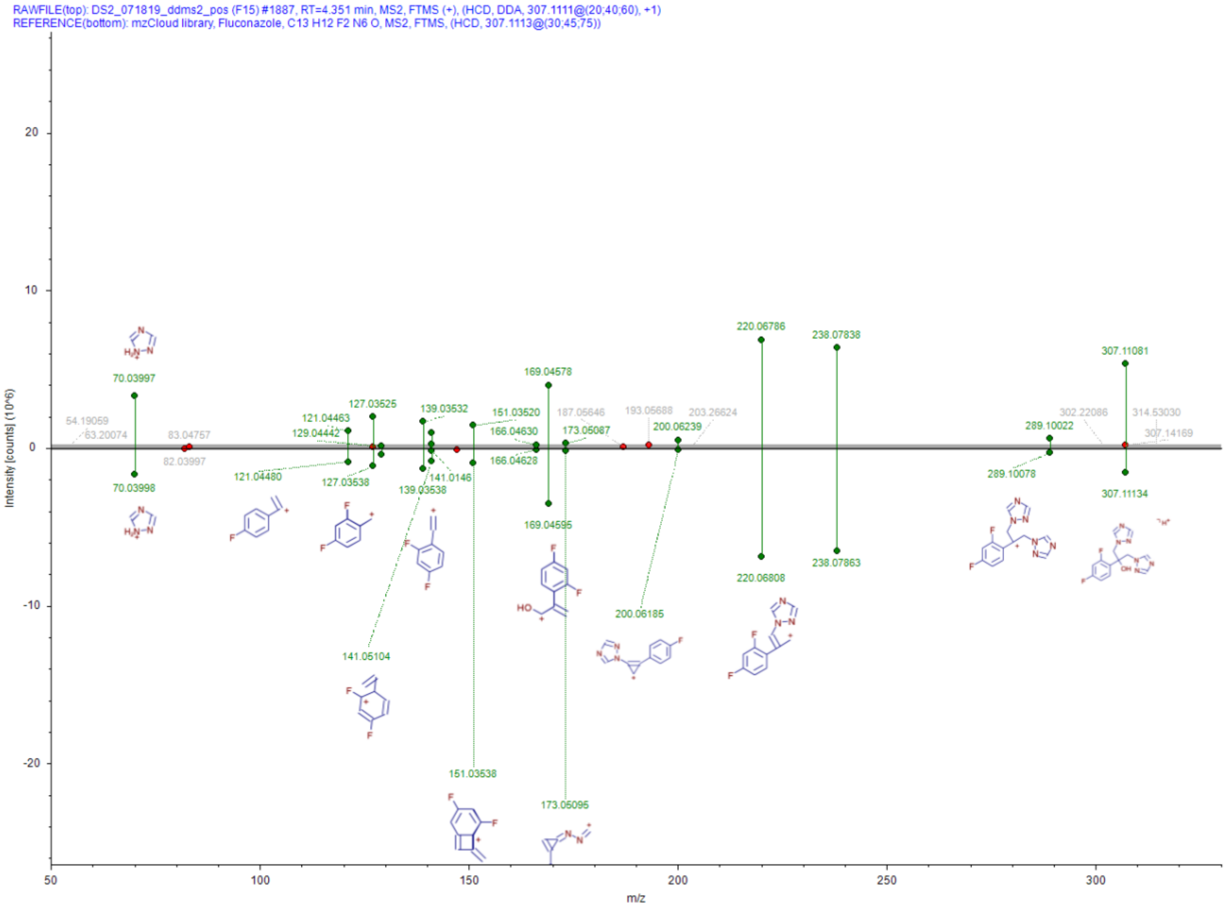

Figure S30. Fluconazole was detected in cluster 4 (DS2). Library spectra matching generated from mzCloud match in Compound Discoverer.

Estrogenicity Results

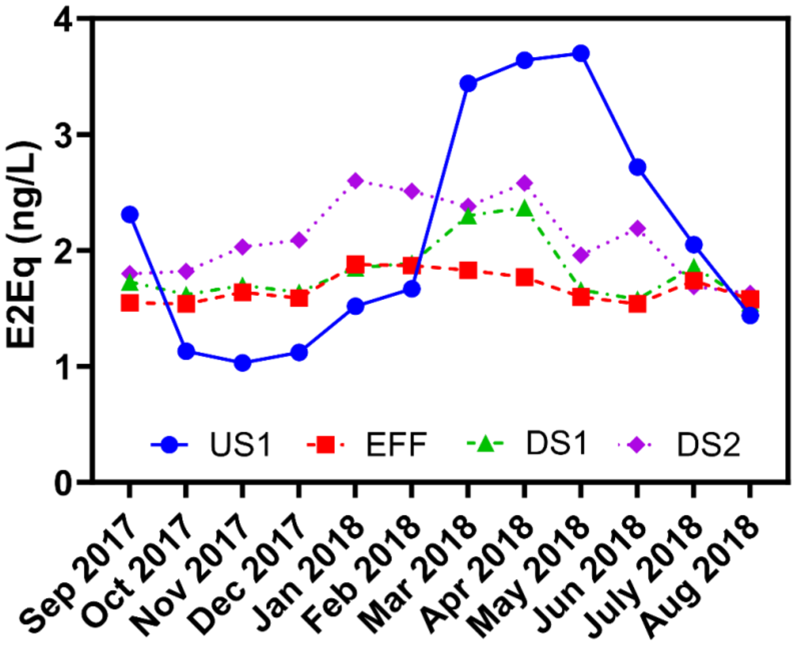

Figure S31. Estrogenicity measured as estrogen equivalents (E_2Eq) were measured monthly at each site in Muddy Creek from September 2017 through August 2018 and reported in a UGGS data release (summarized in Meade et al[4,9]). E_2Eq was the most variable and extreme at US1 compared to the sites directly impacted by the WWTP. Listed concentrations are as follows: US1: 1.03–3.70 ng/L $E_2Eq_{(BLYES)}$; EFF: 1.54–1.88 ng/L $E_2Eq_{(BLYES)}$; DS1: 1.55–2.37 ng/L $E_2Eq_{(BLYES)}$

Samples from US1-January and US1-May (representing respective low and high estrogenicity events in 2018) were processed with Compound Discoverer and filtered leaving only features that had $Log_2FC>2$ and a significance of $p<0.05$ after differential analysis. 302 distinct features were upregulated in US1-May (based on normalized peak area) and a manual filter was then applied to remove compounds with bad peak shape, leaving 109 suspect features of varying confidence. Out of 109 features, 3 only had exact mass hits (Level 5), 20 only had unequivocal molecular formulas (Level 4), and the remaining 85 had assigned names based on predicted compositions, mzCloud, ChemSpider, or MassLists including NORMAN (Level 3). The CompTox Chemicals Dashboard v2.4.1[11] was then used to screen for bioactivity ratings for the

remaining 85 compounds. 56 named compounds were successfully evaluated for bioactivity, and among those, 7 were suspected to be bioactive/estrogenic based on the ToxCast model.[12]

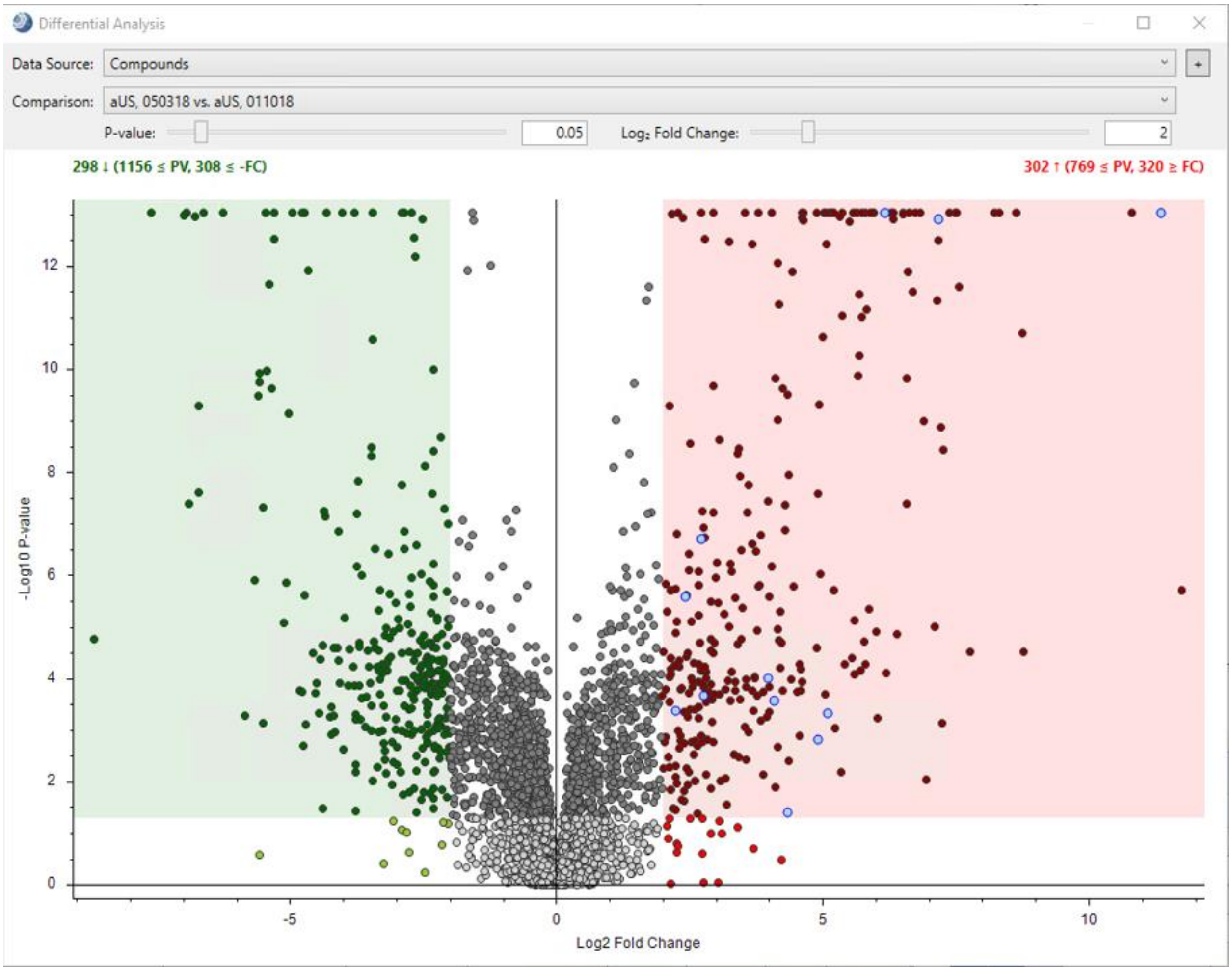

Figure S32. Volcano plot comparing changes in peak area between US1-January and US1-May. Statistical parameters were Log_2 Fold Change = 2 and $p < 0.05$ Green areas represent significantly downregulated compounds, n=298 (more in January than May). Red areas represent significantly upregulated compounds, n=302 (more in May than January). Compounds highlighted in blue have predicted estrogenic activity and are listed in Table S9**Error! Reference source not found.**.

Table S9. Seven suspect compounds with endocrine disrupting potential (as determined with CompTox[11]) were significantly upregulated in US1 – May 2018 ($p < 0.05$, $Log_2FC > 5$), and specifically named and identified (based on MS2 spectra matches from MzCloud librairy match in Compound Discoverer)

Name	Class
Atrazine	herbicide
Siduron	herbicide
1,2-Benzisothiazolin-3-one	antimicrobial
Gentian Violet	dye
19-Norandrosterone (probable match)	androgen
5α-Dihydrotestosterone (probable match)	androgen
Melengestrol (probable match)	estrogen metabolite

‘Head-to-tail’ MS2 spectra matching for estrogenic compounds named in the text

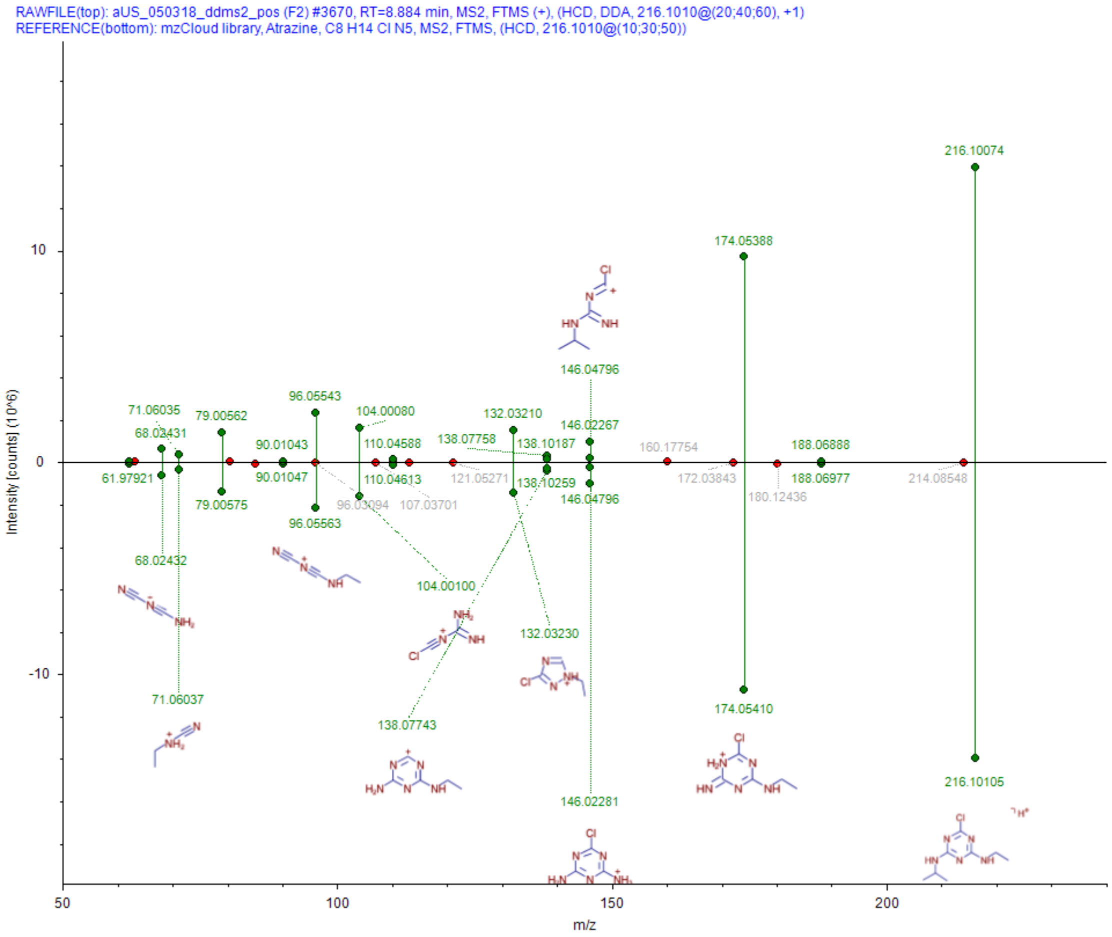

Figure S33. Atrazine (herbicide) heads-tails diagram generated from mzCloud library spectra match in Compound Discoverer. Here, atrazine was upregulated in US1 and has estrogenic properties.

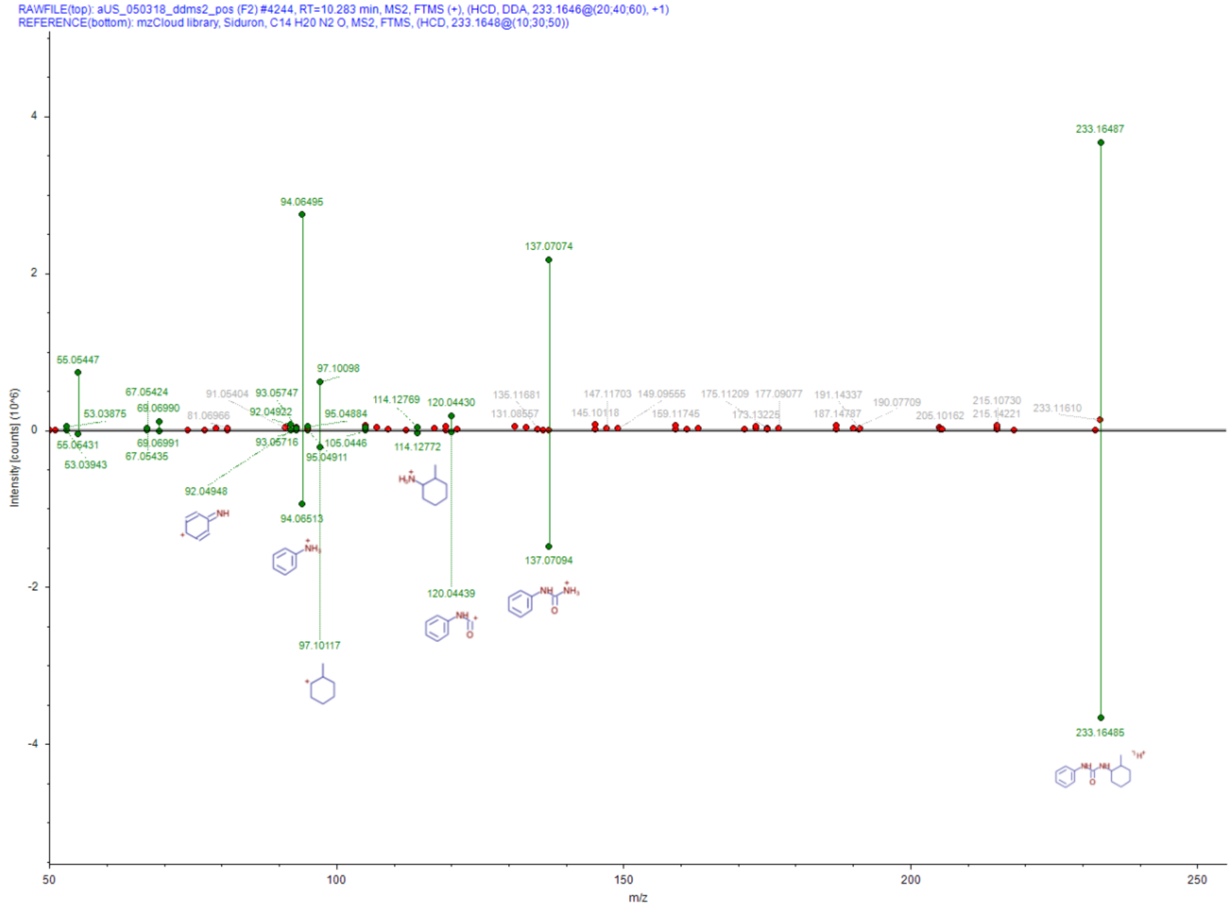

Figure S34. Siduron (herbicide) heads-tails diagram generated from mzCloud library spectra match in Compound Discoverer. Here, siduron was upregulated in US1 and has estrogenic properties.

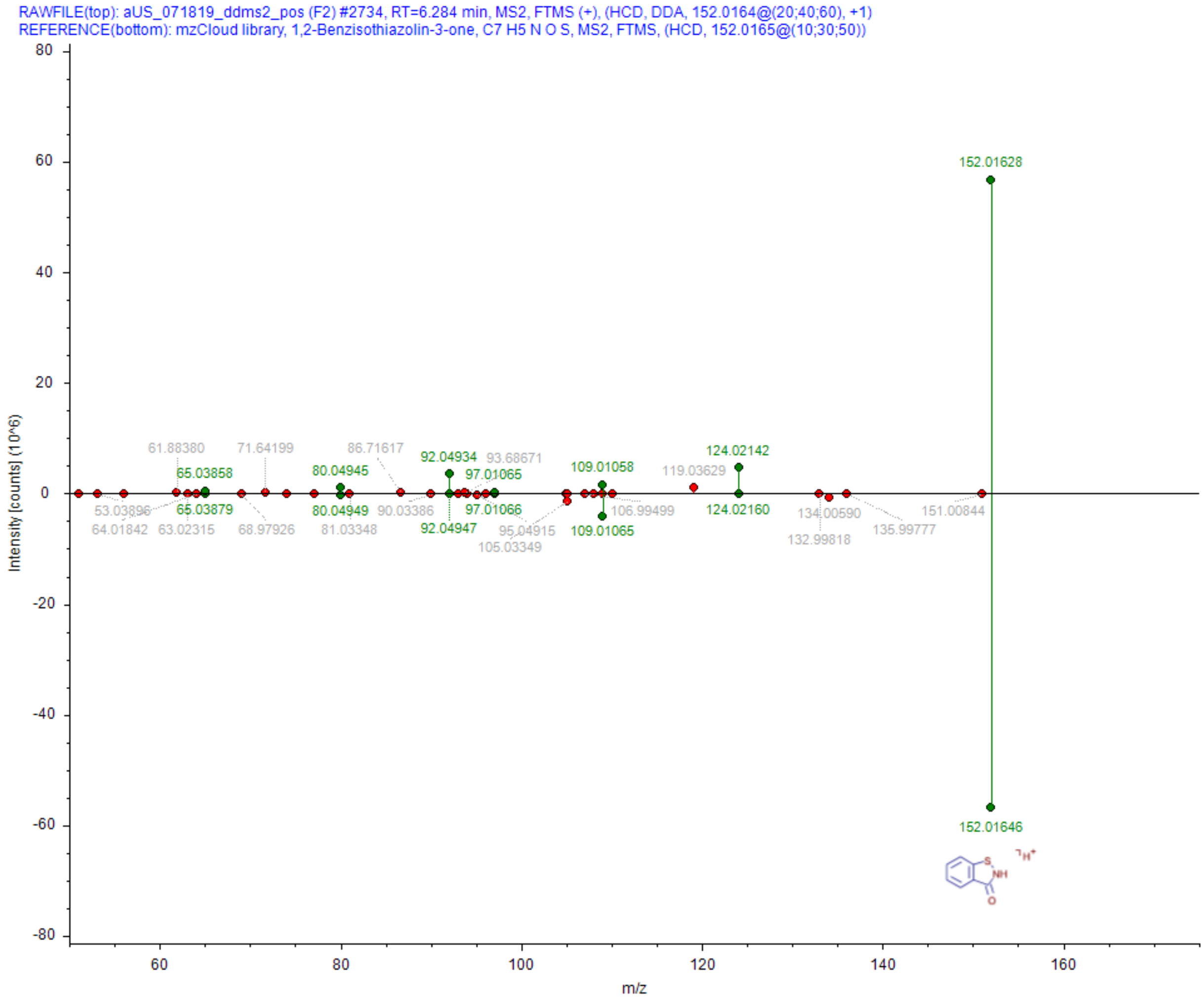

Figure S35. Benzisothiazolinone (antimicrobial also called1,2-Benzisothiazolin-3-one) heads-tails diagram generated from mzCloud library spectra match in Compound Discoverer. Here, 1,2-Benzisthiazolin-3-one was upregulated in US1 and has estrogenic properties.

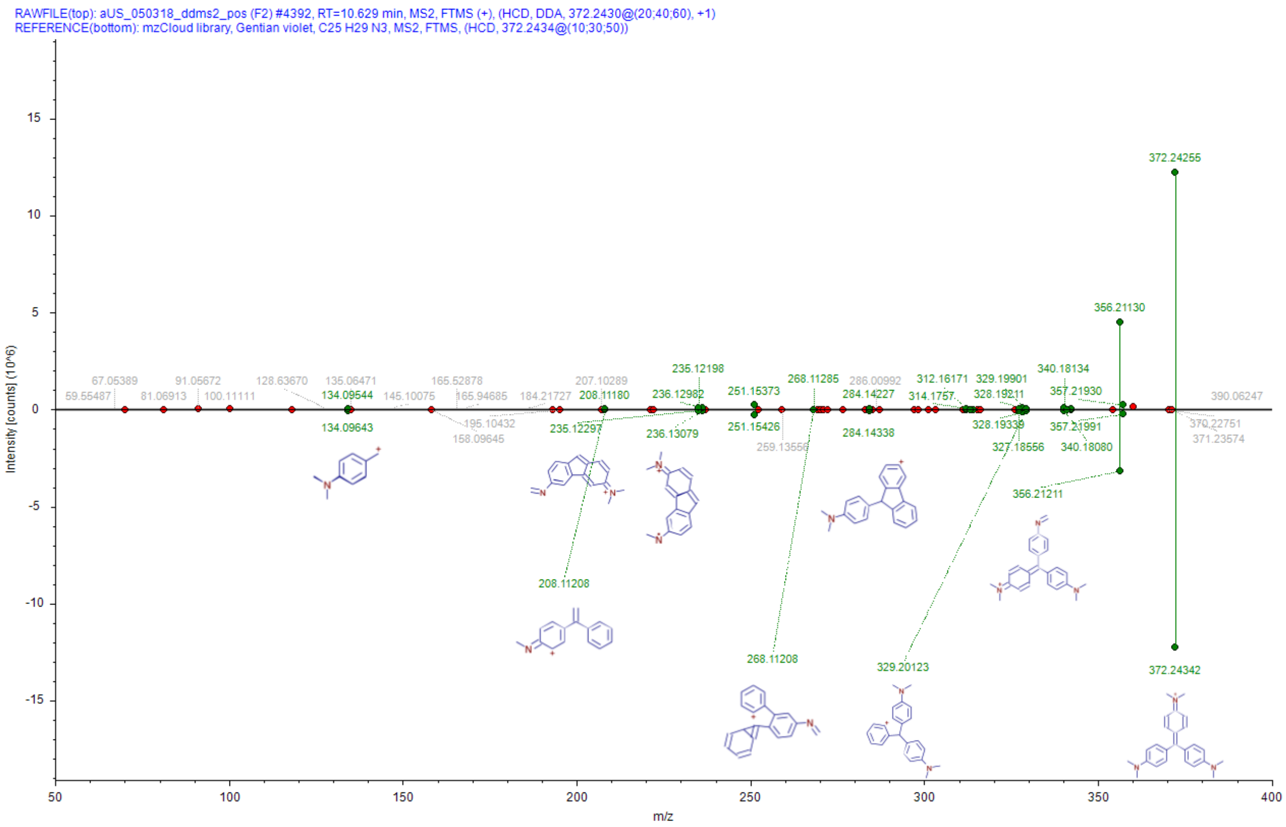

Figure S36. Gentian Violet (dye) heads-tails diagram generated from mzCloud library spectra match in Compound Discoverer. Here, Gentian Violet was upregulated in US1 and has estrogenic properties.

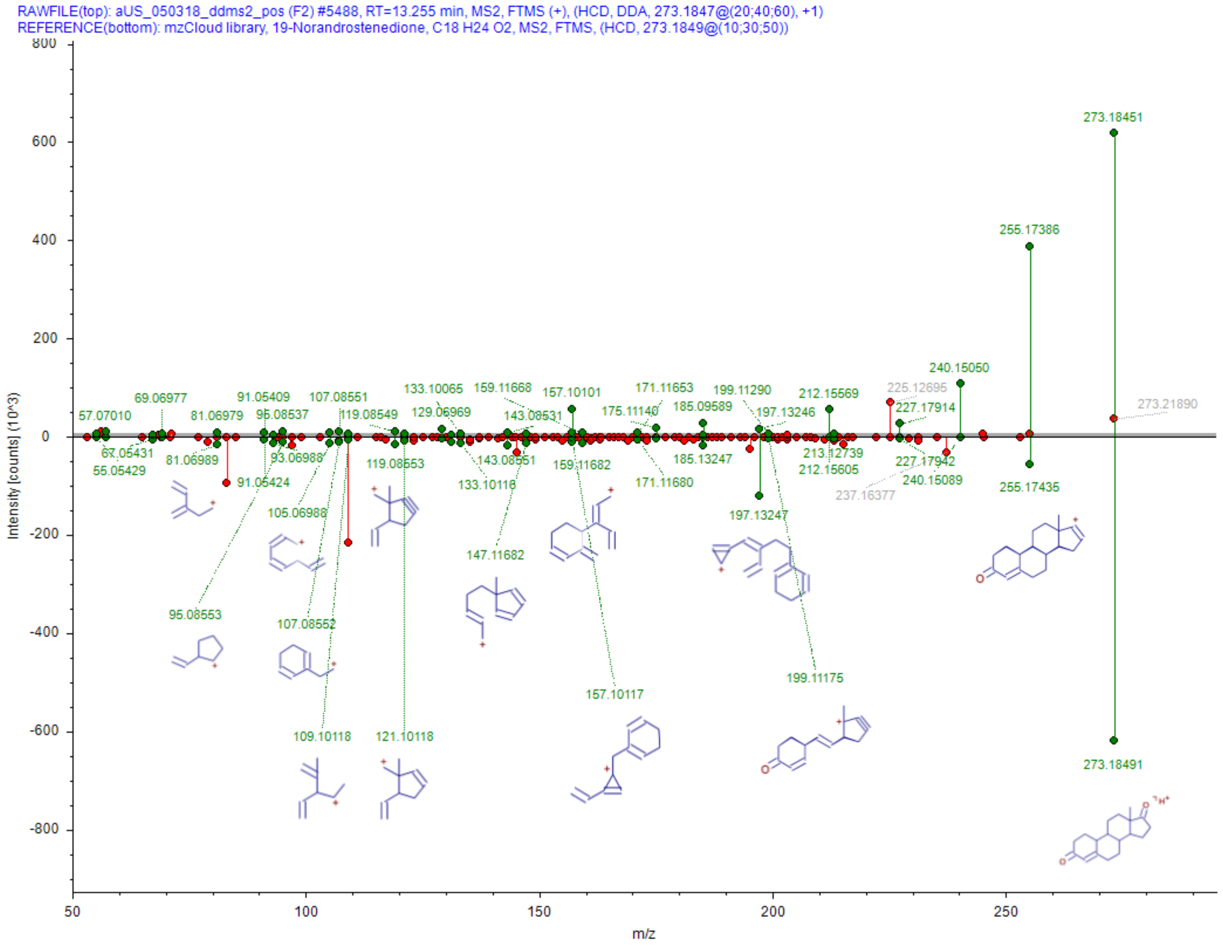

Figure S37. 19-Norandrosterone (androgen type steroid) heads-tails diagram generated from mzCloud library spectra match in Compound Discoverer. Here, 19-Norandrosterone was upregulated in US1 and has estrogenic properties. Many sex steroids are difficult to elucidate from each other using UPLC/HRMS, especially when there are not native standards to compare. Therefore, we are confident that this match is a testosterone type steroid, but we only have probable evidence that it is indeed 19-Norandrosterone.

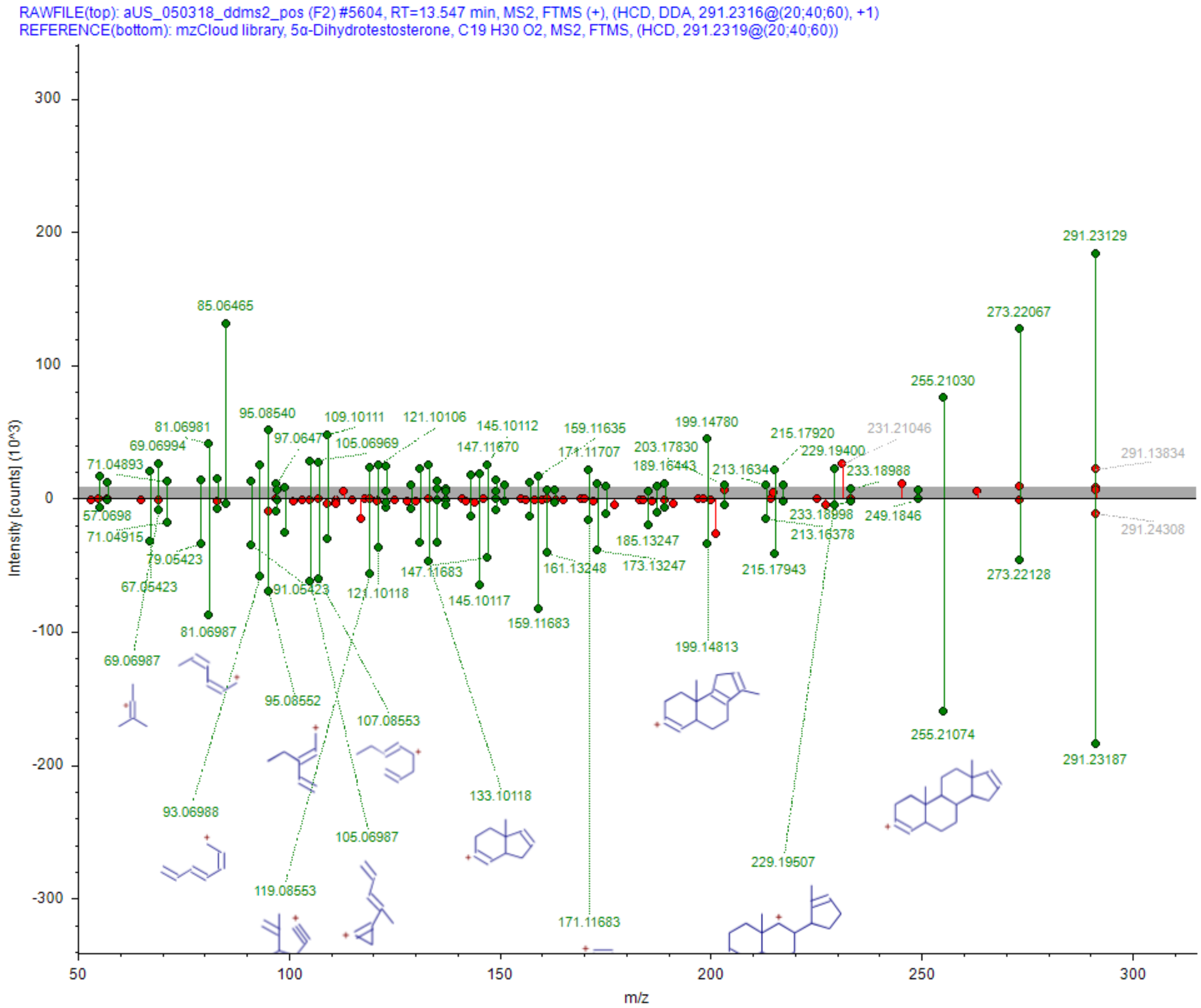

Figure S38. 5α-Dihydrotestosterone (androgen type steroid) heads-tails diagram generated from mzCloud library spectra match in Compound Discoverer. Here, 5α-Dihydrotestosterone was upregulated in US1 and has estrogenic properties. Many sex steroids are difficult to elucidate from each other using UPLC/HRMS, especially when there are not native standards to compare. Therefore, we are confident that this match is a testosterone type steroid, but we only have probable evidence that it is indeed 5α-Dihydrotestosterone.

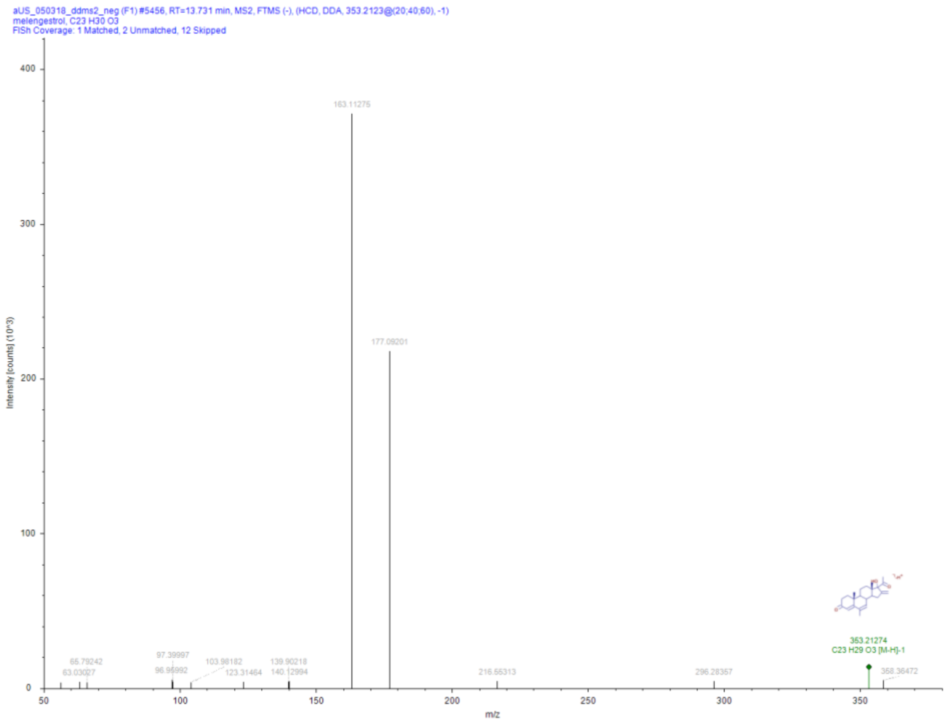

Figure S39. Melengestrol MS2 was generated in Compound Discoverer. No mzCloud library spectra match was available and tentative identification was based on the Compound Discoverer Fragment Ion Search (FISh) tool; thus, identification is weaker than other compounds with a strong mzCloud library match. Here, melengestrol was upregulated in US1 and has estrogenic properties.

Section S5: References

(1) Zhi, H.; Kolpin, D. W.; Klaper, R. D.; Iwanowicz, L. R.; Meppelink, S. M.; Lefevre, G. H. Occurrence and Spatiotemporal Dynamics of Pharmaceuticals in a Temperate-Region Wastewater Effluent-Dominated Stream: Variable Inputs and Differential Attenuation Yield Evolving Complex Exposure Mixtures. *Environ. Sci. Technol* **2020**, *54* (20), 12967–12978. https://doi.org/10.1021/acs.est.0c02328.

(2) Webb, D. T.; Zhi, H.; Kolpin, D. W.; Klaper, R. D.; Iwanowicz, L. R.; LeFevre, G. H. Emerging Investigator Series: Municipal Wastewater as a Year-Round Point Source of Neonicotinoid Insecticides That Persist in an Effluent-Dominated Stream. *Environ. Sci. Process. Impacts* **2021**, *23* (5), 678–688. https://doi.org/10.1039/D1EM00065A.

(3) Furlong, E. T.; Kanagy, C. J.; Kanagy, L. K.; Coffey, L. J.; Burkhardt, M. R. Determination of Human-Use Pharmaceuticals in Filtered Water by Direct Aqueous Injection–High-Performance Liquid Chromatography/Tandem Mass Spectrometry. *B. 5, Lab. Anal.* **2014**, 49. https://doi.org/http://dx.doi.org/10.3133/tm5B10.

(4) Meade, E. B.; Iwanowicz, L. R.; Neureuther, N.; LeFevre, G. H.; Kolpin, D. W.; Zhi, H.; Meppelink, S. M.; Lane, R. F.; Schmoldt, A.; Mohaimani, A.; Mueller, O.; Klaper, R. D. Transcriptome Signatures of Wastewater Effluent Exposure in Larval Zebrafish Vary with Seasonal Mixture Composition in an Effluent-Dominated Stream. *Sci. Total Environ.* **2023**, *856*, 159069. https://doi.org/10.1016/J.SCITOTENV.2022.159069.

(5) Schumann, P. G.; Meade, E. B.; Zhi, H.; LeFevre, G. H.; Kolpin, D. W.; Meppelink, S. M.; Iwanowicz, L. R.; Lane, R. F.; Schmoldt, A.; Mueller, O.; Klaper, R. D. RNA-Seq Reveals Potential Gene Biomarkers in Fathead Minnows (Pimephales Promelas) for

Exposure to Treated Wastewater Effluent. *Environ. Sci. Process. Impacts* **2022**, *24* (10), 1708–1724. https://doi.org/10.1039/D2EM00222A.

(6) Troxell, K.; Ceccopieri, M.; Gardinali, P. Unraveling the Chemical Fingerprint of the Miami River Sources: Insights from High-Resolution Mass Spectrometry and Nontarget Analysis. *Chemosphere* **2024**, *349*, 140863. https://doi.org/10.1016/j.chemosphere.2023.140863.

(7) Mohammed Taha, H.; Aalizadeh, R.; Alygizakis, N.; Antignac, J.-P.; Arp, H. P. H.; Bade, R.; Baker, N.; Belova, L.; Bijlsma, L.; Bolton, E. E.; Brack, W.; Celma, A.; Chen, W.-L.; Cheng, T.; Chirsir, P.; Čirka, Ľ.; D'Agostino, L. A.; Djoumbou Feunang, Y.; Dulio, V.; Fischer, S.; Gago-Ferrero, P.; Galani, A.; Geueke, B.; Głowacka, N.; Glüge, J.; Groh, K.; Grosse, S.; Haglund, P.; Hakkinen, P. J.; Hale, S. E.; Hernandez, F.; Janssen, E. M.-L.; Jonkers, T.; Kiefer, K.; Kirchner, M.; Koschorreck, J.; Krauss, M.; Krier, J.; Lamoree, M. H.; Letzel, M.; Letzel, T.; Li, Q.; Little, J.; Liu, Y.; Lunderberg, D. M.; Martin, J. W.; McEachran, A. D.; McLean, J. A.; Meier, C.; Meijer, J.; Menger, F.; Merino, C.; Muncke, J.; Muschket, M.; Neumann, M.; Neveu, V.; Ng, K.; Oberacher, H.; O'Brien, J.; Oswald, P.; Oswaldova, M.; Picache, J. A.; Postigo, C.; Ramirez, N.; Reemtsma, T.; Renaud, J.; Rostkowski, P.; Rüdel, H.; Salek, R. M.; Samanipour, S.; Scheringer, M.; Schliebner, I.; Schulz, W.; Schulze, T.; Sengl, M.; Shoemaker, B. A.; Sims, K.; Singer, H.; Singh, R. R.; Sumarah, M.; Thiessen, P. A.; Thomas, K. V.; Torres, S.; Trier, X.; van Wezel, A. P.; Vermeulen, R. C. H.; Vlaanderen, J. J.; von der Ohe, P. C.; Wang, Z.; Williams, A. J.; Willighagen, E. L.; Wishart, D. S.; Zhang, J.; Thomaidis, N. S.; Hollender, J.; Slobodnik, J.; Schymanski, E. L. The NORMAN Suspect List Exchange (NORMAN-SLE): Facilitating European and Worldwide Collaboration on Suspect Screening in High

Resolution Mass Spectrometry. *Environ. Sci. Eur.* **2022**, *34* (1), 104. https://doi.org/10.1186/s12302-022-00680-6.

(8) Schymanski, E. L.; Jeon, J.; Gulde, R.; Fenner, K.; Ruff, M.; Singer, H. P.; Hollender, J. Identifying Small Molecules via High Resolution Mass Spectrometry: Communicating Confidence. *Environ. Sci. Technol.* **2014**, *48* (4), 2097–2098. https://doi.org/10.1021/es5002105.

(9) Meppelink, S. M.; Kolpin, D. W.; Lane, R. F.; R. Iwanowicz, L.; Zhi, H.; LeFevre, G. H. Water-Quality Data for a Pharmaceutical Study at Muddy Creek in North Liberty and Coralville, Iowa, 2017-2018. U.S. Geological Survey data release 2020. https://doi.org/https://doi.org/10.5066/P9WOD2XB.

(10) Meppelink, S. M.; Gray, J. L.; Hubbard, L. E.; Cwiertny, D. M.; Thompson, D. A.; Kolpin, D. W. Water-Quality Data for a Statewide Assessment of per- and Polyfluoroalkyl Substances (PFAS) Study in Iowa, 2019-2020. U.S. Geological Survey data release 2021. https://doi.org/https://doi.org/10.5066/P9UJW8GL.

(11) Williams, A. J.; Grulke, C. M.; Edwards, J.; McEachran, A. D.; Mansouri, K.; Baker, N. C.; Patlewicz, G.; Shah, I.; Wambaugh, J. F.; Judson, R. S.; Richard, A. M. The CompTox Chemistry Dashboard: A Community Data Resource for Environmental Chemistry. *J. Cheminform.* **2017**, *9* (1), 1–27. https://doi.org/10.1186/S13321-017-0247-6/FIGURES/15.

(12) U.S. Environmental Protection Agency. ToxCast & Tox21 Summary Files from invitroDBv4.1 database https://www.epa.gov/chemical-research/toxicity-forecaster-toxcasttm-data (accessed Jul 15, 2024).
